# Evidences for Chlorogenic Acid — A Major Endogenous Polyphenol Involved in Regulation of Ripening and Senescence of Apple Fruit

**DOI:** 10.1371/journal.pone.0146940

**Published:** 2016-01-12

**Authors:** Yu Xi, Dai Cheng, Xiangquan Zeng, Jiankang Cao, Weibo Jiang

**Affiliations:** College of Food Science and Nutritional Engineering, China Agricultural University, No. 17 Qinghua Donglu, Beijing 100083, PR China; South China Agricultural University, CHINA

## Abstract

To learn how the endogenous polyphenols may play a role in fruit ripening and senescence, apple pulp discs were used as a model to study the influences of chlorogenic acid (CHA, a major polyphenol in apple pulp) on fruit ripening and senescence. Apple (‘Golden Delicious’) pulp discs prepared from pre-climacteric fruit were treated with 50 mg L^-1^ CHA and incubated in flasks with 10 mM MES buffer (pH 6.0, 11% sorbitol). Compared to the control samples, treatment with CHA significantly reduced ethylene production and respiration rate, and enhanced levels of firmness and soluble solids content of the pulp discs during incubation at 25°C. These results suggested that CHA could retard senescence of the apple pulp discs. Proteomics analysis with sodium dodecyl sulfate-polyacrylamide gel electrophoresis and mass spectrometry (MALDI-TOF/TOF) revealed that the expressions of several key proteins correlated to fruit ripening and senescence were affected by the treatment with CHA. Further study showed that treating the pulp discs with CHA remarkably reduced levels of lipoxygenase, β-galactosidase, NADP-malic enzyme, and enzymatic activities of lipoxygenase and UDP-glucose pyrophosphorylase, all of which are known as promoters of fruit ripening and senescence. These results could provide new insights into the functions of endogenous phenolic compounds in fruit ripening and senescence.

## Introduction

Polyphenols in most of climacteric tree fruits are usually known to be important for their contribution to the taste, colour and nutritional properties of fruits [1]. Previous studies related to biofunctions of fruit polyphenols have focused on their antimicrobial properties, antioxidant properties, bioavailability and bioefficacy in humans [2,3,4]. So far, little is known whether/how endogenous polyphenols may play a role in postharvest ripening and senescence of fruits, despite of markedly changes in phenolic components and content during ripening of various fruits [5,6]. Apple is a kind of typical climacteric fruit and rich in polyphenols [7], and was used as an experimental model in this study to investigate correlations of polyphenols with fruit ripening and senescence. At present, it is difficult to determine endogenous functions of polyphenols in fruit by over-expressing or silencing synthesis of the phenolic compounds in tree-fruits. Thus, discs of fruit pulp have been often used as a model to study biofunctions of various chemicals that are hardly infiltrated through fruit peel into the pulp. It has been demonstrated that excised pericarp discs of tomato fruit could maintain the most whole fruit ripening process compared with intact fruit [8]. The effects of exogenous jasmonates on ethylene biosynthesis and ripening of apple fruit have been determined by treating the apple pulp discs with jasmonates [9].

Chlorogenic acid is a principal component of polyphenols in various climacteric fruits, particularly in tree fruits [10,11,12,13], therefore, it was used as a representative of endogenous-polyphenols, and infiltrated into apple pulp discs to investigate how endogenous polyphenols may influence apple fruit ripening and senescence. Our result demonstrated that chlorogenic acid could suppress apple pulp discs senescence.

## Material and Methods

### 2.1 Plant material

Pre-climacteric apple (*Malus domestica* Borkh. cv. “Golden Delicious”) obtained from a wholesale market in Beijing (China), were selected for uniformity in shape, colour, and size, and then were used for the experiments.

### 2.2 Discs preparation and treatments

Following tests were carried under aseptic condition. The pre-climacteric fruit were sliced crosswise into circular slabs approximately 3 mm thick. The pulp discs prepared from the slabs using an 10 mm diameter cork borer were immersed in 50 mg L^-1^ of chlorogenic acid (CHA), or distilled water (as control) in desiccators, then vacuumed (-0.02 M Pa) for 1 min at 25°C. Thereafter, the discs (5 pieces, about 3 g) were placed on the sterile filter paper at bottom of a 100 mL flask containing 3 ml of 10 mM MES buffer (pH 6.0, 11% sorbitol), and incubated at 25°C. For measuring ethylene production and respiration rate, gas samples were taken at the indicate times from the flask sealed for 2 h. Samples were taken at the indicated times for firmness, soluble solids content (SSC) or for the other analysis being stored at -80°C. Chlorogenic acid (3-O-caffeoylquinic acid) were from Fluka-Sigma-Aldrich (St. Louis, MO, USA).

### 2.3 Extraction of polyphenols and analysis with HPLC

5.0 g of frozen sample was ground in a mortar, then transferred into a capped centrifuge tube with 20 ml of 80% ethanol. The mixture was sonicated for 45 min, then centrifuged at 10,000*×g* at 4°C for 30 min. The supernatant (polyphenol extract) was collected, evaporated to dryness under vacuum at 30°C, then dissolved in 5 mL of deionized water and stored at -20°C.

HPLC analysis was performed using Shimadzu LC-20AT pumps, SPD-M20A diode array detection, and chromatographic separations were performed on a C18 column (Shim-pack VP-ODS 15 cm×4.6 mm ID, 5 μm, Shimadzu, Japan). The mobile phase consisted of 1% (v/v) acetic acid in water (eluent A) and methanol (eluent B). According to Liu et al. [14], the eluting gradient was programmed as follows: 12–25% B (0–15 min), 25–35% B (15–25 min), 35–55% B (25–50 min), 55–65% B (50–60 min), and 65–12% B (60–70 min). Operating conditions were as follows: 35°C column temperature, 10 μL injection volume and UV-diode array detection at 280 nm.

### 2.4 Assay of apple discs quality parameters

Respiration rate was determined by gas chromatography as described by Alique and Zamorano [15]. Ethylene production was determined by gas chromatography as described by Jiang et al. [16]. Results of respiration rate were expressed as μl CO_2_ g^-1^ h^-1^. Results of ethylene production were expressed as μl C_2_H_4_ g^-1^ h^-1^. Flesh firmness of apple discs was determined by using a Fruit Firmness Tester (GY-2, Tuopu Instrument Co., Ltd., Zhejiang, China) with a 3.5 mm probe. SSC was determined using the same methods as previously reported [17].

### 2.5 Protein extraction and polyacrylamide gel electrophoresis (PAGE)

Briefly, 1.0 g of frozen sample was finely powdered in a mortar with liquid nitrogen and then homogenized with 100 μL ice-cold 1 M Tris (pH 11.2) and 30 mg PVPP. The homogenate was centrifuged at 10,000×*g* for 30 min, at 4°C and the supernatant was collected. Then the supernatant was put in dialysis solution (2 mM Tris-HCl, pH 7.5) overnight at 4°C. The protein after dialysis was twenty-fold concentrated and centrifuged at 12,000×*g* for 20 min, at 4°C and the supernatant was collected. The protein content was determined by Bradford method [18], using bovine serum albumin (Sigm-Aldrich Chemical Co., St. Louis, MO, USA) as standard.

Native—polyacrylamide gel electrophoresis (PAGE) was performed with a 10% separating gel and a 4% stacking gel. The electrophoresis were run under non-reducing conditions. Apple protein samples were mixed with 1 time the volume of a non-reducing loading buffer [100 mM Tris–HCl, pH 6.8, 20% (v/v) glycerol, 0.01% bromophenol blue], and 30 μL of mixed sample were loaded in each lane with 45 μg of protein. Protein bands were stained in 0.1% (w/v) R 250 Coomassie Brilliant Blue and distained in a solution of 10% (v/v) ethanol and 10% (v/v) acetic acid.

For SDS (sodium dodecylsulfate)-PAGE analysis, protein samples were dissolved in loading buffer containing 200 mM pH 6.8 Tris-HCl, 2% SDS, 10% β-mercaptoethanol and run on a 4% (w/v) stacking gel and 10% (w/v) separate gel. Individual native-PAGE apple protein bands were separated by SDS-PAGE under the same conditions. After R 250 Coomassie Brilliant Blue staining and distaining, native-PAGE bands were excised horizontally, washed with deionized water three times for 10 min to remove ethanol and acetic acid, and then vibrated with 1% SDS for 30 min before SDS—PAGE. Protein samples with the sample buffer [100 mM Tris-HCl, pH 6.8; 20% (v/v) glycerol, 2% (v/v) SDS, 5% (v/v) b-mercaptoethanol and 0.01% bromophenol blue] at a 1:1 ratio (v/v) were heated in boiling water for 5 min, and then centrifuged at 10,000×*g* for 2 min. The supernatant was run on SDS-PAGE. Molecular weight standards, specifically SDS-PAGE standards (Thermo Fisher Scientific Co., Waltham, MA, USA), with molecular weights ranging from 25 kDa to 170 kDa were used. The intensities were quantified using Quantity One software (Bio-Rad Co., Hercules, CA, USA).

### 2.6 Protein in—gel digestion and identification by MALDI-TOF/TOF

The protein in-gel digestion was accordding to Zhang et al. [19] with some changes. Protein bands were excised from the gels and washed with double-distilled water and then transferred to sterilized Eppendorf tubes. Then the protein bands were distained with 50 mM NH_4_HCO_3_ in 50% ethanol for 2 h at 40°C. The proteins therein were then reduced with 10 mM DTT in 50 mM NH_4_HCO_3_ and alkylated with 55 mM iodoacetamide in 50 mM NH_4_HCO_3_ for 1 h at room temperature. The proteins were digested overnight at 37°C by adding 15 μL of trypsin (Promega Co., Madison, WI, USA). The resulting peptides were extracted by washing the gel pieces with 0.1% trifluoroacetic acid in 67% ACN. Tryptic peptide masses were analyzed by a 4700 MALDI-TOF/TOF Proteomics Analyzer (Applied Biosystems, Carlsbad, CA, USA). Proteins were identified by searching against the NCBInr *Malus domestica* (apple) and *Rocaseace* database using an in-house MASCOT server v 2.1 (Matrix Science Co., London, UK).

### 2.7 Raising polyclonal antibodies and immune blot analysis

New Zealand white rabbits were initially injected with 300 μg of purified protein in Freund’s complete adjuvant (Sigm-Aldrich Chemical Co., St. Louis, MO, USA) followed by a booster injection of 300 μg of the same immuogen in Freund’s incomplete adjuvant (Sigm-Aldrich Chemical Co., St. Louis, MO, USA) 21 days later, then the rabbits were injected with 300 μg of the same immuogen in Freund’s incomplete adjuvant (Sigm-Aldrich Chemical Co., St. Louis, MO, USA) 14 days later. After 10 days, blood was collected and IgG antibodies were purified as described by Biggs et al.[20]. All experimental protocols were approved by the Animal Management Rules of the Ministry of Health of the People’s Republic of China (documentation Number 55, 2001, Ministry of Health of PR China), with utilization permission from Animal Department of Academy of Military Medical Sciences, No. SCXK (Jun) 2007–004. All surgery was performed under anesthesia, and all efforts were made to minimize suffering. For serum preparation, the rabbits were anaesthetized with diethyl ether and blood samples were collected from the auricular vein of the rabbits.

Immune-analysis by Western-blot was carried to examine expression of the proteins related to senescence in apple fruit following the method of Wang et al.[21]. The proteins (20 μg per lane) were separated with 10% SDS-PAGE and transferred onto nitrocellulose membranes. Subsequently, the membranes were blocked for 2 h with 5% skimmed milk and then incubated with rabbit polyclonal antibody raised against β-galactosidase, NADP-malic enzyme and thaumatin-like protein at 1:5000 dilutions for 2 h. The membrane was washed (3 times for 15 min) with TBST buffer (0.01 M TBS, 0.1% Tween-20, pH 7.6) and then incubated with a secondary goat anti—rabbit IgG conjugated with horseradish peroxidase at 1:8000. Immunoblot signals were detected with ECL (Boster Co., Wuhan, China).

### 2.8 Assay of lipoxygenase and UGPase

Lipoxygenase (LOX) extraction and assay. 2.0 g of frozen sample were ground in ice-cold mortar and pestle with 1 mL 0.5 M Tris (pH 7.8). The homogenate was centrifuged at 10,000×*g* for 30 min at 4°C. The supernatant was collected and used for analysis. LOX activity was determined according to Gökmen et al. [22]. One unit of enzyme activity is defined as the amount of enzyme producing one unit change in absorbance per minute at 234 nm at 25°C. Enzyme activity was expressed as U g^-1^ on a fresh-weight basis.

UDP-glucose pyrophosphorylase (UGPase) extraction and assay. 2.0 g of frozen sample was homogenized in a mortar and pestle with 6 mL of buffer consisting of 100 mM HEPES, pH 7.5, 5 mM MgCl_2_, 1 mM EDTA, 2 mM GSH, 0.1% Na_2_HSO_3_ (w/v) and 1% PVP (w/v). After a 10 min grinding period, the suspensions were centrifuged at 10,000×*g* for 15 min. The supernatant was collected and used for analysis. Activity of UGPase was assayed using the one-step method previously described [23]. One unit of enzyme activity is defined as the amount of enzyme producing one unit change in absorbance per minute at 340 nm at 25°C. Enzyme activity was expressed as U g^-1^ on a fresh-weight basis.

### 2.9 Statistical analysis

Data were evaluated by the analysis of variance (ANOVA) with Statistical Analysis System (SAS version 9.2, SAS Institute Inc., NC, US, 2003). Significant differences were performed by Duncan’s new multiple range tests, where differences at *p* < 0.05 were considered as significant.

## Results

### 3.1 Phenolic compounds in apple pulp and effects of CHA on apple discs senescence

**Fig 1:**
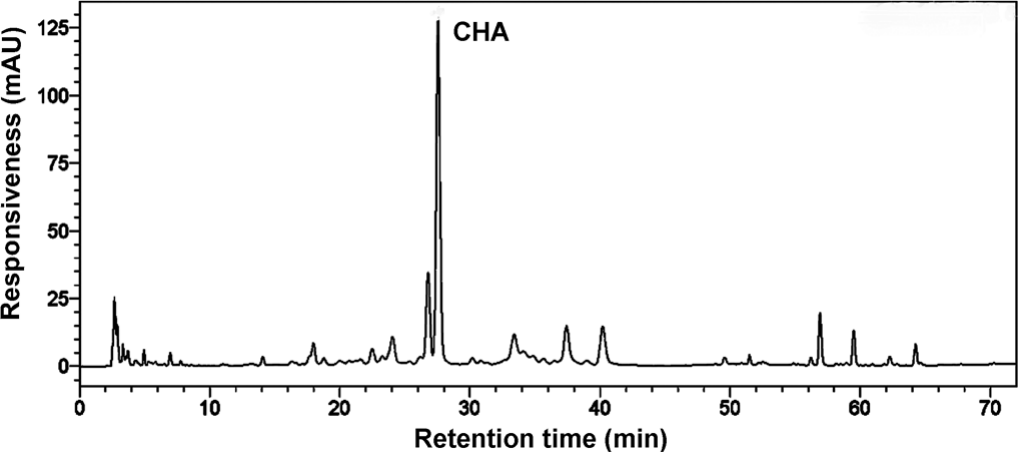
HPLC-chromatographic profile of phenolic compounds in apple pulp at 280 nm wavelengths. The major peak was identified by both of internal standard method and external reference method with chlorogenic acid (3-O-caffeoylquinic acid, CHA) as a standard substance.

Our analysis with HPLC showed that chlorogenic acid (3-O-caffeoylquinic acid, CHA) was a major phenolic compound in the apple pulp. CHA content in the pulp was 54 mg kg^-1^ FW, and took about 49% of total polyphenols content in pulp (Fig 1). Thus, CHA (50 mg L^-1^) was used as a representative endogenous-polyphenol, and infiltrated into apple pulp discs to evaluate effects of endogenous polyphenols on fruit senescence.

**Fig 2:**
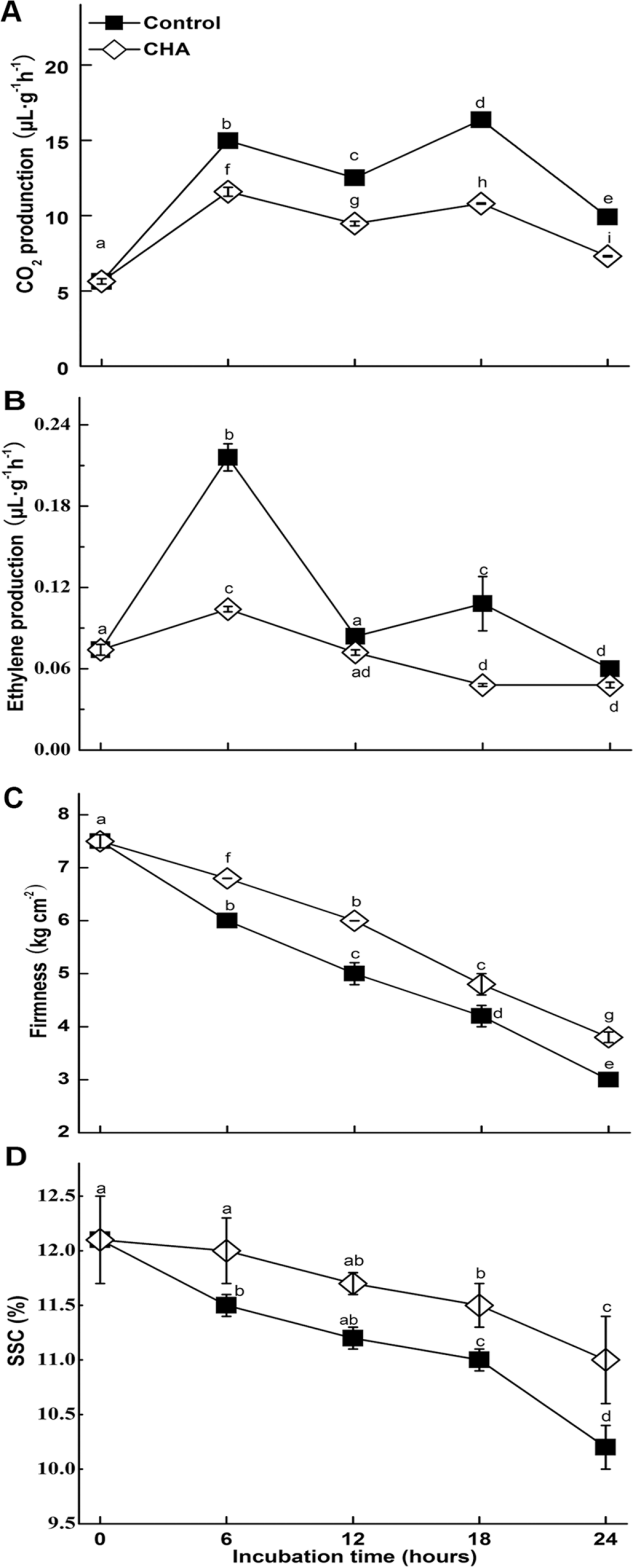
Effects of CHA on apple pulp discs incubated at 25°C. Each data point represents a mean ± standard deviation (for respiration rates and ethylene production, n = 3; for firmness and SSC, n = 6). Values with different letters are significantly different at *p* < 0.05.

Ethylene synthesis and respiration rate of the discs increased during incubation, and both of which were significantly reduced by treatment with CHA. As shown in Fig 2A, respiration rate of control samples increased 165% or 198% after 6 h or 18 h of incubation; meanwhile, respiration rate of CHA-treated discs was only 78% or 66% of that in control, respectively. Similarly, ethylene production of control samples increased 183% or 46% after 6 h or 18 h of incubation; ethylene production of CHA-treated discs was only 48% or 44% of that in control, respectively (Fig 2B).

Treatment with CHA also remarkably retarded decreases of firmness and SSC of the apple pulp discs during the incubation (Fig 2C and 2D).

### 3.2 Identification of proteins related to senescence of apple pulp discs and analysis of mass spectrometry

**Fig 3:**
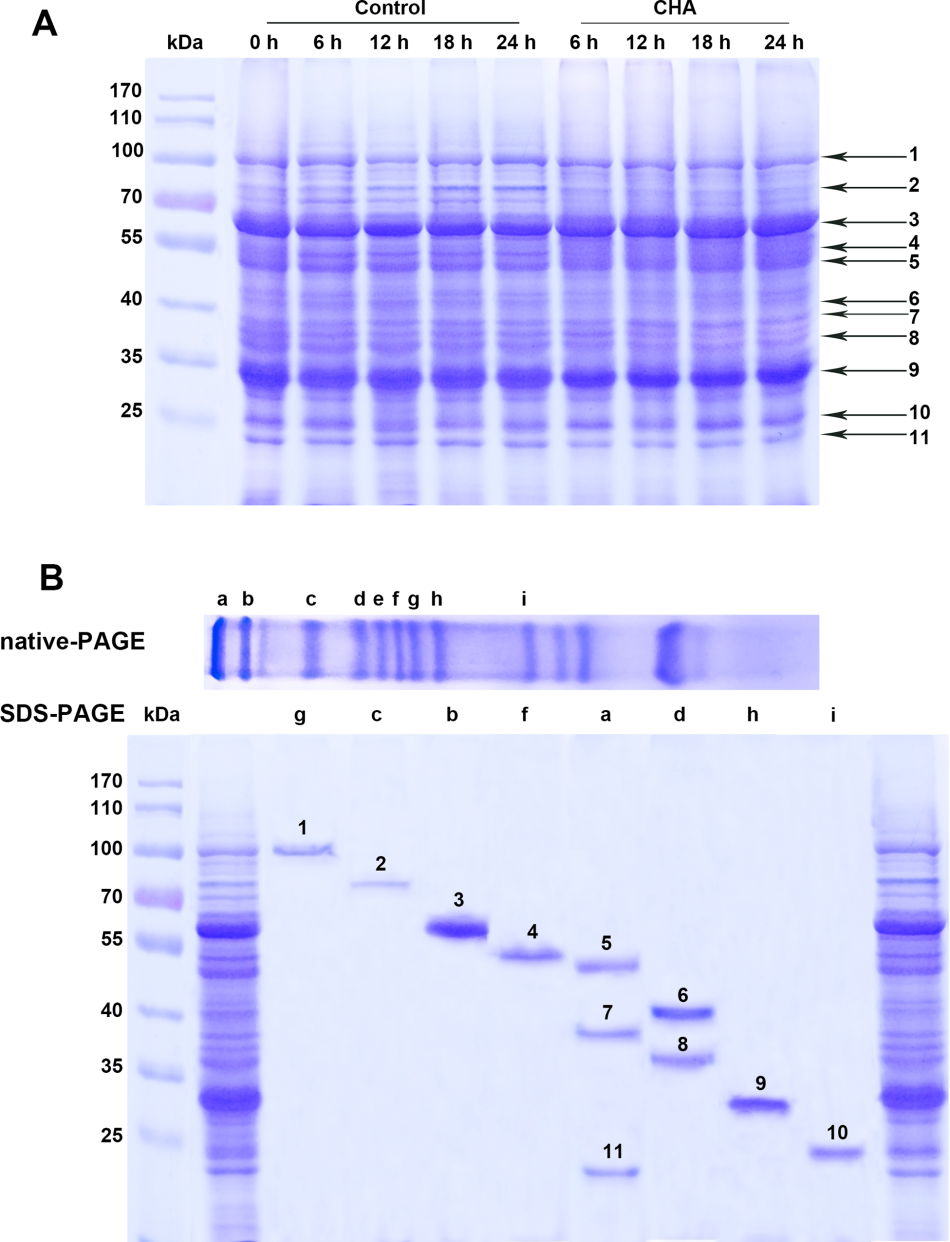
Identification of proteins related to senescence of apple pulp disc. (A) SDS-PAGE analysis of protein profile of apple pulp discs during incubation at 25°C; (B) Identifying and isolation of proteins (band 1–11) related to senescence of apple pulp discs by native-PAGE and SDS–PAGE.

In SDS-PAGE analysis of protein profile of the apple pulp discs, 11 major protein bands were found to be correlated with fruit ripening and senescence (Fig 3A). These proteins present a Mr from 20 kD to 120 kD. We further found that resolution of the proteins was much higher in native-PAGE than in SDS-PAGE, therefore protein extract of the pulp discs was first separated by native-PAGE, then the major bands were isolated and re-run on SDS-PAGE. Thereafter, the 11 proteins excised from the gel of SDS-PAGE were submitted for analysis of mass spectrometry (MALDI-TOF/TOF, data in S1 Appendix.).

The identification results and a complete list of the protein sequences with peptides delivered by mass spectra were presented in Table 1, Table 2. According to Clusters of Orthologous Groups of proteins (COG, http://www.ncbi.nlm.nih.gov/COG/), six of them were involved in metabolic process (protein 1, 2, 3, 5, 6,7), three of them were involved in response to stress (protein 4, 8, 9); one was catalogued in biological process (protein 10); one was catalogued in transport (protein 11).

**Table 1:** Senescence related proteins in apple pulp discs identified by MALDI-TOF/TOF.

No.	protein name^a^	source^b^	accession^c^	Mr	pI	score	Coverage^d^
**1**	lipoxygenase	*Malus domestica*	gi 471328166	97854	5.14	153	30%
**2**	b-galactosidase	*Malus domestica*	gi 507278	81628	5.63	232	50%
**3**	NADP-malic enzyme	*Fragaria vesca subsp*. *vesca*	gi 470102042	65317	6.32	96	26%
**4**	dehydrin COR47	*Malus domestica*	gi 658033116	31783	5.20	297	37%
**5**	UDP-glucosepyrophosphorylase	*Pyrus pyrifolia*	gi 3107931	51984	5.99	83	45%
**6**	PREDICTED: phosphoglycerate kinase, cytosolic	*Malus domestica*	gi 657982239	42392	6.36	170	39%
**7**	glutamine synthetase cytosolic isozyme	*Malus domestica*	gi 658036616	39094	5.78	650	52%
**8**	thaumatin-like protein precursor	*Malus domestica*	gi 3643249	26609	5.17	160	31%
**9**	thaumatine-like protein	*Malus domestica*	gi 394986175	24108	4.72	113	34%
**10**	5'-3' exoribonuclease 2	*Malus domestica*	gi 657979046	22112	5.74	86	18%
**11**	ferritin-4, chloroplastic	*Malus domestica*	gi 657992077	29414	5.44	252	35%

a: proteins identified by MALDI-TOF/TOF

b: species the matched proteins from

c: protein accession from NCBInr

d: matched ammonia acid coverage.

**Table 2:** Peptide sequences identified from the apple pulp by mass spectrometry and their localization within matched proteins.

**1 lipoxygenase**				
MLHNLLGKT	GQQQDGESNI	GK**INGTVVLM**	**KKNVLDFNDF**	**NASVLDRVHE**
**LVGQR**VSLQL	ISAVHGDPDN	GLKGNLGKQA	YLEDWITTIT	PLTAGESAFK
VTFDWEEEVG	VPGAFIIQNN	HHSEFFLK**TV**	**TLDNVPDEGR**	VHFVCNSWVY
PAEKYTKDRV	FFANK**TYLPS**	**EVPLPLRKYI**	**EEELVELRGD**	**GKGKLEEWDR**
VYDYAYYNDL	GDPDKGSEYV	RPIMGGSTEY	PYPRRGRTGR	PPKETDPNTE
SR**LPIVSSLS**	**IYVPR**DERFG	HLKMSDFLAY	ALKSIAQFIR	PEIEALFDKT
PNEFDSFKDV	LQLYEGGIPL	PEGLFKEIGD	SIPAEMLKEI	FR**TDGAQFLR**
**FPMPEVIK**VD	KTAWRTDEEF	AREMLAGVNP	VNIRLLQEFP	PASKLDPK**VY**
**GDQTSTITEQ**	**HIR**NNLDGLT	VDEALKNKKL	FILDHHDALM	PYLR**RINSTS**
**NK**IYGSR**TLL**	**FLKSDGTLK**I	LVIELSLPHP	DGDQYGCISN	VYTPAEQGVE
SSIWQLAKAY	VAVNDSGNHQ	LISHWLNTHA	VIEPVIIAAN	R**QLSVVHPIY**
**KLLQPHFRDT**	**MYINAIGRGI**	**LLNARGVIES**	**TVFPAR**YALG	LSSAVYKDWI
FPEQALPADL	IKR**GVAVKDE**	**NSPHGLR**LLI	EDYPYAVDGI	EIWFAIKTWV
EDYCAFYYKT	NEIIQTDVEL	QSWWK**ELVEE**	**GHGDIKDEPW**	**WPK**MQTFEEL
VETCTILIWT	ASALHAALNF	GQFSYAGYLP	NRPTISRKFM	PEK**GTPEYEE**
**LEASPDTVFL**	**K**TITAQLQTV	LGIATIEILS	R**HSTDEVYLG**	**QR**DTPDWTSD
TAALEAFDKF	GKKLAEIEDR	**ITSMNNDEKL**	**K**NRVGSVKIP	YTLLFPTSEG
GITGKGIPNS	VSI			
**2 β-galactosidase**				
MGVGIQTMWS	ILLLFSCIFS	AASASVSYDH	KAIIINGQK**R**	**ILISGSIHYP**
**RSTPEMWPDL**	**IQK**AKDGGLD	VIQTYVFWNG	HEPSPGNYYF	EERYDLVKFI
KLVQQEGLFV	NLRIGPYVCA	EWNFGGFPVW	LK**YVPGIAFR**	TDNEPFKAAM
QKFTEKIVSM	MKAEK**LFQTQ**	**GGPIILSQIE**	**NEFGPVEWEI**	**GAPGK**AYTKW
AAQMAVGLDT	GVPWIMCKQE	DAPDPVIDTC	NGFYCENFKP	NKDYKPKMWT
EVWTGWYTEF	GGAVPTRPAE	DVAFSVAR**FI**	**MYHGGTNFGR**	**QSGGSFLNYY**
**TAGGPFMATS**	**YDYDAPLDEY**	**GLPREPK**WGH	LRDLHKAIK**S**	**CESALVSVDP**
**SVTKLGSNQE**	**AHVFKSESDC**	**AAFLANYDAK**	YSVK**VSFGGG**	**QYDLPPWSIS**
**ILPDCK**TEVY	NTAKVGSQSS	QVQMTPVHSG	FPWQSFIEET	TSSDETDTTT
LDGLYEQINI	TR**DTTDYLWY**	**MTDITIGSDE**	**AFLK**NGKSPL	LTIFSAGHAL
NVFINGQLSG	TVYGSLENPK	**LSFSQNVNLR**	SGINKLALLS	ISVGLPNVGT
HFETWNAGVL	GPITLK**GLNS**	**GTWDMSGWKW**	**TYKTGLKGEA**	**LGLHTVTGSS**
**SVEWVEGPSM**	**AEKQPLTWYK**	**ATFNAPPGDA**	**PLALDMGSMG**	**KGQIWINGQS**
**VGRHWPGYIA**	**RGSCGDCSYA**	**GTYDDKK**CR**T**	**HCGEPSQRWY**	**HIPRSWLTPT**
**GNLLVVFEEW**	**GGDPSRISLV**	**ERGTALDAK**K	L	
**3 NADP-malic enzyme**				
MDSTLKEMRD	GVSALDLDSK	SAVGGGVEDI	YGEDAATEDQ	LVTPWTYSVA
SGYSLLRDPQ	YNK**GLAFTEK**	**ERDAHYLR**GL	LPPATSSQEL	QEKKLMHNLR
**QYQVPLQKYM**	**ALTELQER**NE	RLFYK**LLIDN**	**VEELLPIVYT**	**PTVGEACQK**Y
GSIFR**RPQGL**	**YISLK**EKGRI	LEVLKNWPER	TIQVIVVTDG	ER**ILGLGDLG**
**CQGMGIPVGK**	LALYTALGGV	RPSTCLPITI	DVGTNNEQLL	KDEFYIGLRQ
KRATGKEYAE	LLHEFMGAVK	QNYGEK**VLVQ**	**FEDFANHNAF**	**ELLAK**YGTTH
LVFNDDIQGT	AAVVLAGVVA	ALKLISGTLS	EHK**FLFLGAG**	**EAGTGIAELI**
**ALEISK**KTKI	PVEETRKK**IW**	**LVDSK**GLIVS	SRKESLQHFK	KPWAHEHEPV
KDLIDAVK**AI**	**KPTVLIGSSG**	**VGR**TFTKEVI	EALASFNEKP	LILALSNPTS
QSECTAEEAY	TWTKGRAIFA	SGSPFDPVEY	NGKVYVPGQS	NNAYIFPGLG
LGLVISGAIR	VHDDMLLAAS	EALAGQVTKE	NIDNGLIYPP	FSKIRKISAA
IAANVAAKAY	ELGVATR**LPR**	**PENLVK**HAES	CMYSPLYRSY	R
**4 dehydrin**				
MAEEYNK**KSD**	**EHEYERKTGD**	**YEEGSGAGET**	**KDRGLFDFLG**	**KKEEEKPTPY**
**QQGDQVNVAE**	**FDEK**VK**ISDH**	**HDQHASSYNK**	VEEEEDKEK**K**	**HETLLQK**LHR
SESSSSSSSD	EEEDEEKKKK	RKEKKGLTDK	IKEKISGDEH	KEEGYHKEED
TAVPVEKVYE	EEHHHPAPAP	APVVHYHEEP	TDSPTEEK**KG**	**FLEK**IKEKLP
GHKKTEEVPV	GAASHEQHSD	DKHAAEPPVA	ASYEAGEEPK	EKKGILEKIK
**EKLPGYHSKT**	**EEDHKDIK**EK	EKDTPSY		
**5 UDP-glucose pyrophosphorylase**				
MAAVATGNVD	KLKSDVASLS	QISENEKNGF	INLVSR**YVSG**	**EEAQHVEWSK**
**IQTPTDEVVV**	**PYDGLAPTPE**	**DPEEIKK**LLD	KLVVLKLNGG	LGTTMGCTGP
KSVIEVRNGL	TFLDLIVIQI	ENLNNKYGSC	VPLLLMNSFN	THDDTQKIVE
KYSK**SNVQIH**	**TFNQSQYPRL**	**VVEDFSPLPS**	**K**GQTGK**DGWY**	**PPGHGDVFPS**
**LK**NSGKLDLL	LSQGK**EYVFI**	**ANSDNLGAVV**	**DLK**ILHHLIQ	KK**NEYCMEVT**
**PKTLADVKGG**	**TLISYEGRVQ**	**LLEIAQVPDQ**	**HVNEFK**SIEK	FKIFNTNNLW
VNLNAIKRLV	EADALKMEII	PNPK**EVDGVK**	**VLQLETAAGA**	**AIR**FFNHAIG
INVPRSRFLP	VK**ATSDLLLV**	**QSDLYTLQDG**	**FVTR**NSARKN	PENPTIELGP
EFKKVGSYLS	RFKSIPSILE	LESLK**VSGDV**	**WFGAGVVLKG**	**K**VTITAKSGV
K**LEIPDNAVI**	**ANK**DINGPED	L		
**6 phosphoglycerate kinase**				
MATKKSVSTL	KEAELKGKRV	FVR**VDLNVPL**	**DDNSNITDDT**	**R**IRAAVPTIK
**YLLGHGAKVI**	**LASHLGRPK**G	VTPK**YSLKPL**	**VPRLSELLGL**	**EVK**IANDCIG
EEVEK**LVAQL**	**PEGGVLLLEN**	**VR**FYKEEEKN	DPEFAK**KLAS**	**LADVYVNDAF**
**GTAHRAHAST**	**EGLAKYLKPS**	**VAGFLMQKEL**	**DYLVGAVSNP**	**KRPFAAIVGG**
**SK**VSTKIGVI	ESLLAKVNVL	LLGGGMIFTF	YK**AQGHSVGS**	**SLVEEDKLDL**
**AK**SLLEKAKS	KGVSILLPTD	VVIADKFAAD	ANSKVVPASA	IPDGWMGLDI
GPDSIKTFSE	ALDTTQTIIW	NGPMGVFEFE	KFAAGTEAIA	KKLAELSGKG
VTTIIGGGDS	VAAVEKAGLA	EKMSHISTGG	GASLELLEGK	TLPGVLALDD
A				
**7 glutamine synthetase**				
MSLLTDLINL	DLSGSTK**KII**	**AEYIWIGGSG**	**MDIR**SKAR**TL**	**PGPVSDPSKL**
**PKWNYDGSST**	**GQAPGEDSEV**	**ILYPQAIFK**D	PFRR**GNNILV**	**ICDTYTPGGE**
**PIPTNKR**AAA	AK**IFSHPDVV**	**AEVPWYGIEQ**	**EYTLLQK**DVX	WPLGWPVGGY
PGPQGPYYCA	AGADKAFGR**D**	**IVDSHYK**ACL	YAGINISGIN	GEVMPGQWEF
QVGPSVGISA	GDELWAARYI	LERITEIAGV	VLSFDPKPIQ	GDWNGAGAHT
NYSTKSMRED	GGYEXIKK**AI**	**DKLGLRHKEH**	**IAAYGEGNER**	**R**LTGR**HETAD**
**INTFK**WGVAN	RGASIR**VGRE**	**TEQAGKGYFE**	**DRRPASNMDP**	**YVVTSMIAET**
**TILLKP**				
**8 thaumatin-like protein precursor**				
MMKSQVAPRP	TLAILFFFSG	AHAAKITFTN	NCPNTVWPGT	LTGDQKPQLS
LTGFELASKA	SR**SVDAPSPW**	**SGR**FWGR**TRC**	**STDAAGK**FTC	ETADCGSGQV
ACNGAGAVPP	ATLVEITIAA	NGGQDYYDVS	LVDGFNLPMS	VAPQGGTGEC
KPSSCPANVN	K**VCPAPLQVK**	AADGSVISCK	**SACLAFGDSK**	**YCCTPPNNTP**
**ETCPPTEYSE**	**IFEKQCPQAY**	**SYAYDDK**NST	FTCSGGPDYV	ITFCP
**9 thaumatin-like protein**				
AKITFTNNCP	NTVWPGTLTG	DQKPQLSLTG	FELASKASR**S**	**VDAPSPWSGR**
FWGRTRCSTD	AAGKFTCETA	DCGSGQVACN	GAGAVPPATL	VEITIAANGG
QDYYDVSLVD	GFNLPMSVAP	QGGTGECKPS	SCPANVNK**VC**	**PAPLQVKAAD**
**GSVISCKSAC**	**LAFGDSKYCC**	**TPPNNTPETC**	**PPTEYSEIFE**	**KQCPQAYSYA**
**YDDK**NSTFTC	SGGPDYVITF	C		
**10 5'-3' exoribonuclease 2**				
MSEEKHHRGL	FHHHKDEDKP	SDYPQSGYSD	EGRPGGLGGG	YGDTNDYSGE
GRTGGLGGGY	GDTNAYSGEG	RPGGYGGYNE	TTAYSEER**VE**	**RPGGGR**YSET
TAAYGSTTTH	ESELDYKKEE	KHHK**HLEHLG**	**EAGVAAAGAF**	**ALHEK**HNEK**K**
**DPEHAHR**HKI	EEEIAAAAAV	GSGGFAFHEH	HEKKETKEEE	EEAYGKKKHH
HF				
**11 ferritin-4**				
MMSLRAISTF	SVPSKLGDKG	GAVTTLLPNS	KLGSSSSTLS	FKPQRKLEKF
AASVSSEAVA	LTGVVFQPFE	EVK**NDAFVVP**	**VSPQVSLAR**Q	RYTDESEAAI
NEQINVEYNV	SYVYHALFAY	FDRDNVALK**G**	**LANFFKESSE**	**EEREHAEKLM**
**EYQNKR**GGRV	KLHSVIAXPT	EFDHAEK**GDA**	**LYAMELALSL**	**EK**LTNEK**LLN**
**LHK**VADQNND	PQLMDFIESE	FLAEQVEAIK	**KIADYVTQLR**	**R**VGK**GHGVWH**
**FDQYLLHEGD**	**AAN**			

A compendium of the protein sequences containing the peptide sequences (bold parts) delivered by the mass spectra is presented.

### 3.3 Effects of CHA on changes in content and enzymatic activity of the major proteins related to senescence of apple pulp discs

**Fig 4:**
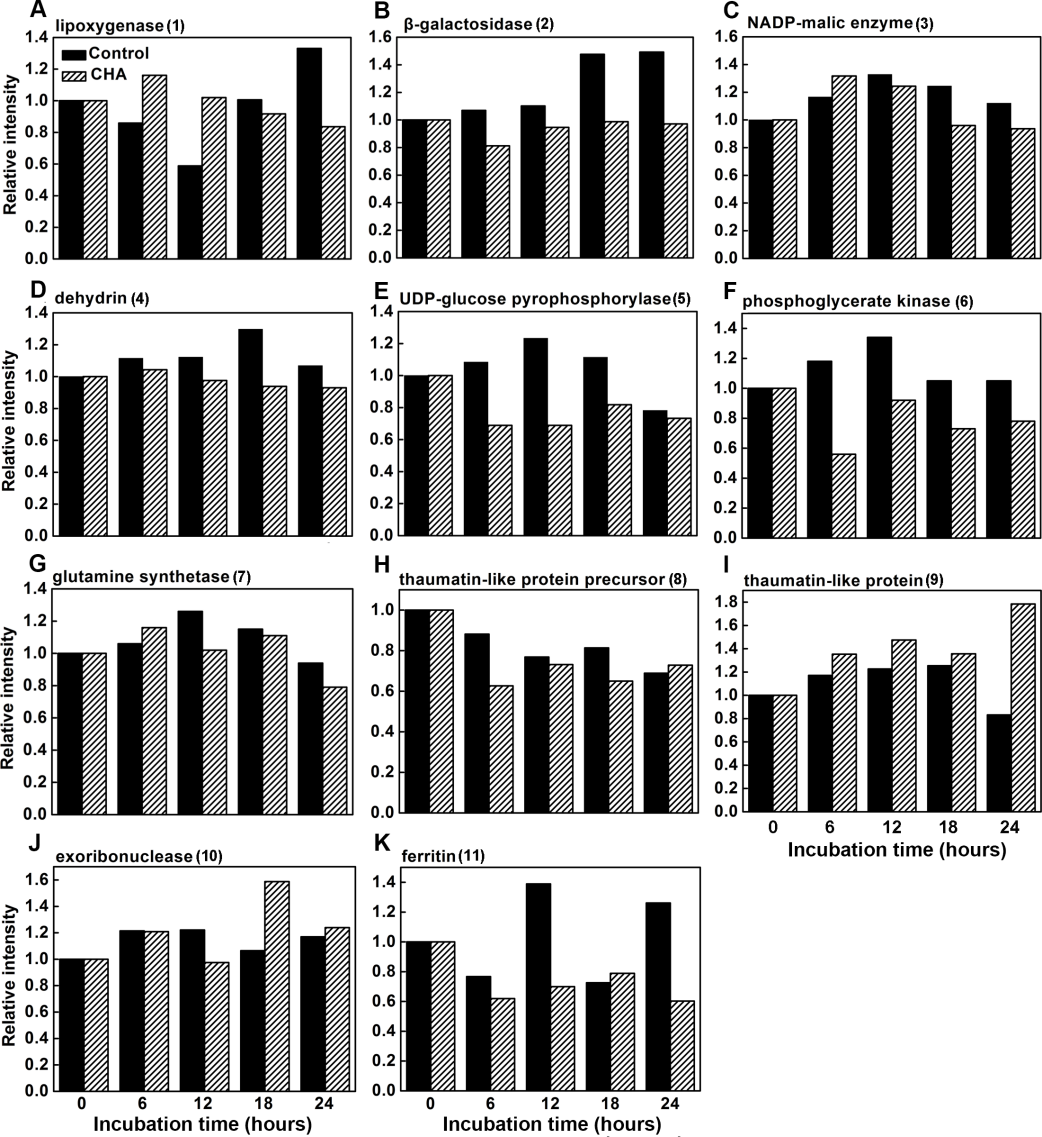
Effect of CHA on changes in levels of the senescence related proteins in apple pulp discs during incubation at 25°C.

As presented in Fig 3A and Fig 4, acumulation levels of lipoxygenase (LOX, band 1), β-galactosidase (β-GAL, band 2), NADP-malic enzyme (NADP-ME, band 3), Dehydrin COR47 (band 4) and ferritin-4 (band 11) increased during the incubation, and were remarkably reduced by CHA-treatment. Meanwhile, levels of thaumatin-like protein (TLP, band 9) and 5'-3' exoribonuclease 2 (band 10) were enhanced by CHA-treatment.

**Fig 5:**
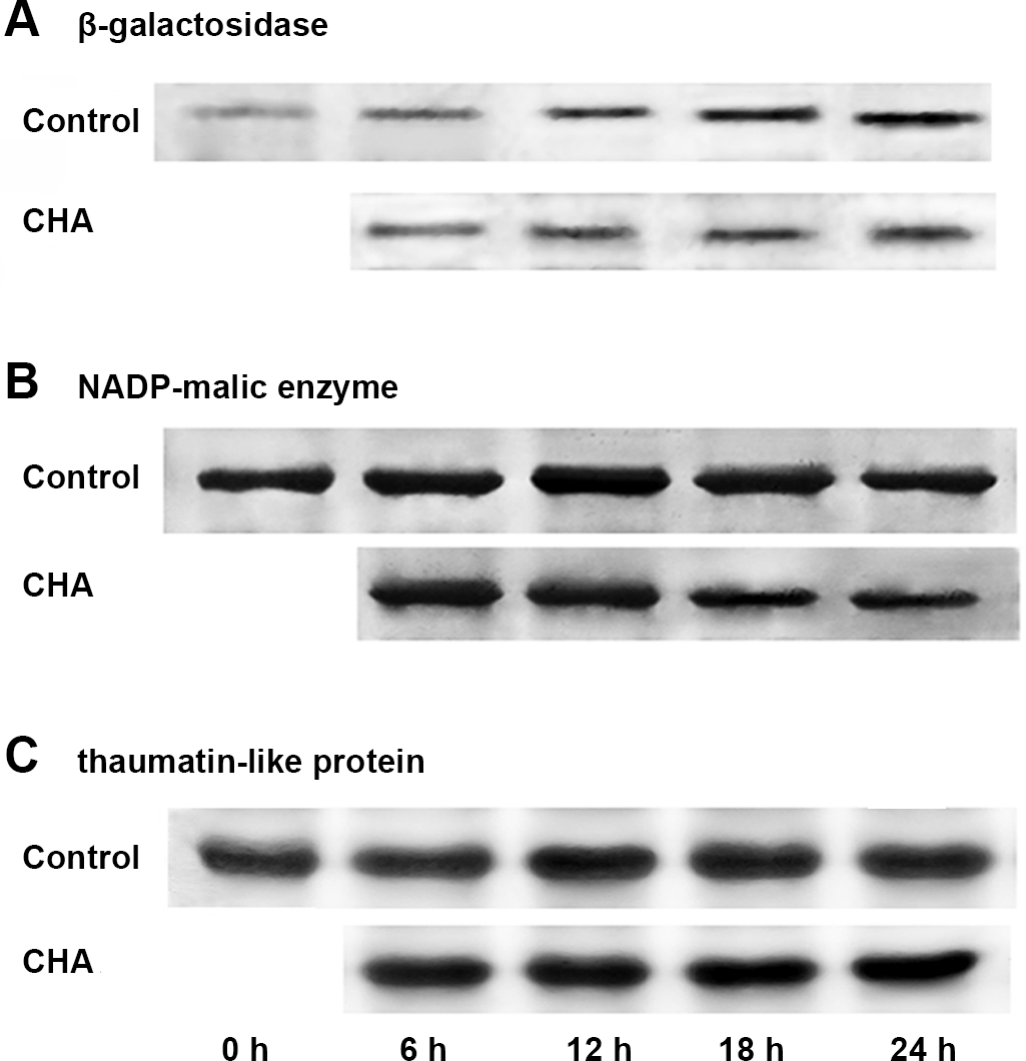
Western blot analysis of (A) galactosidase; (B) NADP-malic enzyme; and (C) thaumatin-like protein in apple pulp discs during incubation at 25°C.

The CHA induced decreasing in protein levels of β-GAL, NADP-ME, and increasing in level of TLP were further demonstrated by immune-blot analysis with antibody against each of the protein respectively (Fig 5).

**Fig 6:**
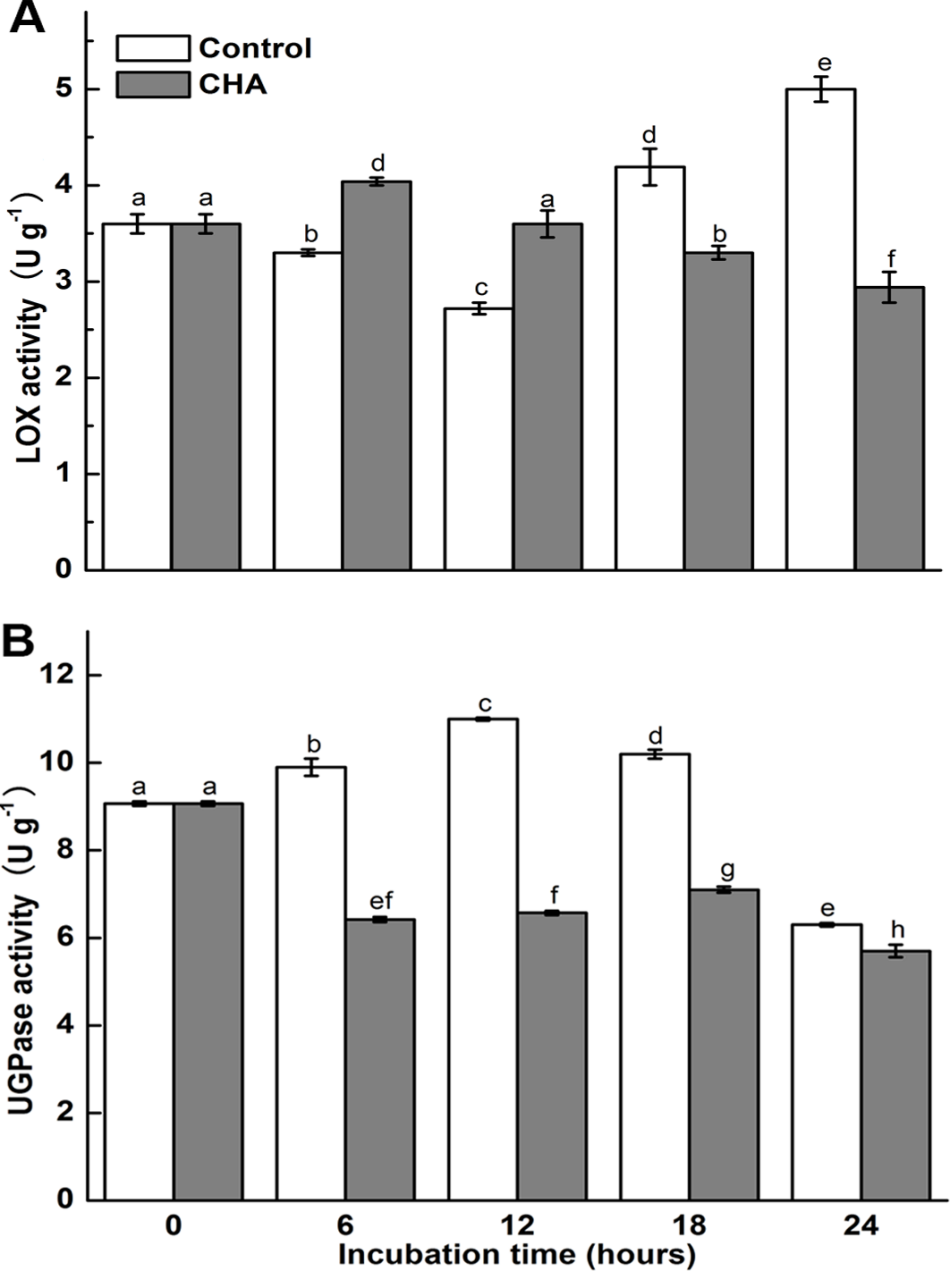
Effects of CHA on activities of lipoxygenase(LOX) and UDP-glucose pyrophosphorylase (UGPase) in apple pulp discs during incubation at 25°C. Each data point represents a mean ±standard deviation (n = 3). Values with different letters are significantly different at *p* < 0.05.

Enzymatic analysis showed that activities of LOX and UDP-glucose pyrophosphorylase (UGPase) in apple pulp were reduced significantly by the CHA-treatment (Fig 6).

## Discussion

Previous studies of apple polyphenols primarily focused on the difference of all cultivars [10,11,12,13], phenolic composition and changing of antioxidants during storage [24,25], or its effects on human disease [26]. Although we noticed that treatment with apple phenolic extract can effectively reduce browning and loss of red colour of litchi pericarp [27], information for physiological roles of endogenous phenolic compounds in fruits is limited. As CHA is the principle phenolic compound in apple pulp, we chose it as a representative compound to research how endogenous-polyphenol were involved in apple fruit senescence. Previous study showed that physiological processes of ripening in tissues of intact fruit could be examined in excised pulp discs [8], so we chose apple pulp discs as a model.

Respiration rate is generally a good indicator of the metabolic rates of harvested fruits and its control can be an effective mean of regulating general metabolism and delaying fruit senescence [28]. In this study, we observed that CHA could inhibit increase in respiration rate of apple pulp discs during senescence. In addition, NADP-ME was found to be inhibited by CHA (Figs 3A, 4C and 5). Considering NADP-ME was implicated in respiration during ripening, providing pyruvate and/or NADPH as a substrate for respiration in fruits, such as tomatoes and grape berries [29,30], we suppose that inhibition of NADP-ME could account for CHA suppressing respiratory rate of apple pulp discs.

After being harvested, although growth has ceased, changing in the composition and organization of pectin, hemicellulose and cellulose polysaccharides of the cell wall and cell-to-cell separation is very pronounced during fruit ripening and is thought to be a key ripening-associated metabolic event that determines the timing and extent of loss of cell adhesion, which leads to fruit softening [31]. Thus firmness is a critical character of postharvest fruits. Reports show that β-GAL plays an important role in fruit softening, like mango, pear, and peach [32,33]. We found that compared with control samples, levels of β-GAL during the discs senescence could be remarkably reduced by CHA (Fig 3A, Fig 4B, Fig 5), these may account for the CHA effect of enhancing firmness of apple pulp discs during the observation.

LOX is known as being responsible for the typical breakdown of linolenic acid, and thus responsible for some physiological disorders [34], and being a major contributor to senescence-related membrane deterioration in a number of plant tissues [35]. In agreement with Cai et al. [36], we observed that LOX increased in apple pulp discs during senescence. Meanwhile, we found CHA could reduce LOX level and activity in apple discs (Figs 3A and 6A), which should also contribute to suppressing apple discs senescence.

UGPase is a major glycosyl donor for polysaccharides in all organisms [37,38], and is believed to be involved in sucrose synthesis in plants [39]. In this study, we found UGPase increased, but SSC decreased in apple pulp discs during the incubation; while CHA-treatment inhibited UGPase accumulation, activity and enhanced SSC level (Figs 2D, 3A, 4E and 6B). Thus, the UGPase might not be the key enzyme related to SSC in apple discs.

This study showed that TLP content in apple pulp discs increased in CHA-treated samples (Figs 3 and 4I). Since TLPs are responsive to biotic and abiotic stress and have antifungal activity [40], therefore, it is reasonable to deduce that CHA and the TLPs could have coordinate effects on antifungal diseases. Although there is no direct evidence to support this hypothesis, the primary sub-cell location of TPLs, same as of CHA, has been found in vacuole [41].

Dehydrins are a family of plant proteins typically induced in response to stress conditions that cause cellular dehydration, such as low temperatures, high salinity, and drought. Dehydrins are known to be important for cell survival during stress [42,43], however, its physiological function in fruit during ripening and senescence is not clear presently. We observed that dehydrin COR 47 increased in control samples, whereas it almost remained the same protein expression level in CHA-treated apple pulp discs during the incubation. This result may suggest that because of reducing senescence of the pulp discs, the stress level was less in CHA-treated discs than that of control samples, therefore, accumulation of dehydrin protein in the CHA-treated discs was also reduced.

The specific functions of 5'-3' exoribonuclease 2 and ferritin-4 during fruit ripening were still not clear; further studies are necessary to confirm whether these two proteins are involved in the CHA-effects on fruit senescence.

## Conclusion

Using apple pulp discs as an experimental model, we observed that CHA, a major endogenous polyphenol in apple fruit, can retard the senescence of apple pulp discs by reducing ethylene production and respiration rate, maintaining firmness and SSC levels. Further study showed that treatment with CHA remarkably reduced levels of lipoxygenase, β-galactosidase, NADP-malic enzyme, and enzymatic activities of lipoxygenase and UDP-glucose pyrophosphorylase, all of which are known as promoters of fruit ripening and senescence. These results provide new insights into the functions of endogenous phenolic compounds in fruit ripening and senescence.

## Supporting Information

S1 Appendix Identification data of proteins listed in Table 1. (DOC)
