# Supplementary material for: Evidences for Chlorogenic Acid — A Major Endogenous Polyphenol Involved in Regulation of Ripening and Senescence of Apple Fruit
Source: PLoS One. 2016 Jan 12;11(1):e0146940. doi: 10.1371/journal.pone.0146940 (PMC4710503; doi:10.1371/journal.pone.0146940)
Supplement: S1 Appendix — (DOC) [file pone.0146940.s001.doc]

**S1 Appendix. Identification data of proteins listed in Table 1.**

**Lipoxygenase:**

**Mass-spectrum (MS/MS):**

**
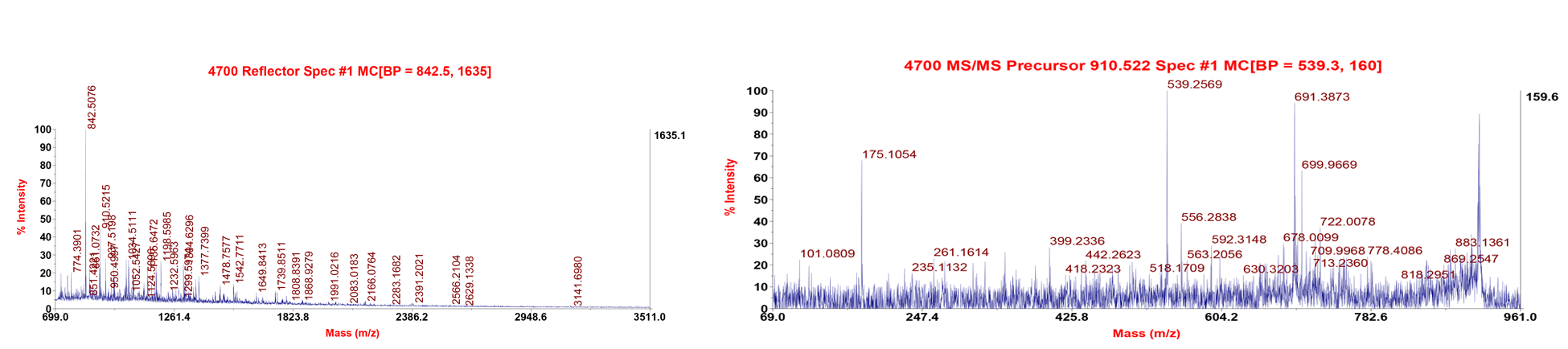
**

**Identification:**

**gi|471328166 Mass: 97854 Score:153 Expect: 3.5e-012 matches: 28**

lipoxygenase [Malus domestica]

**
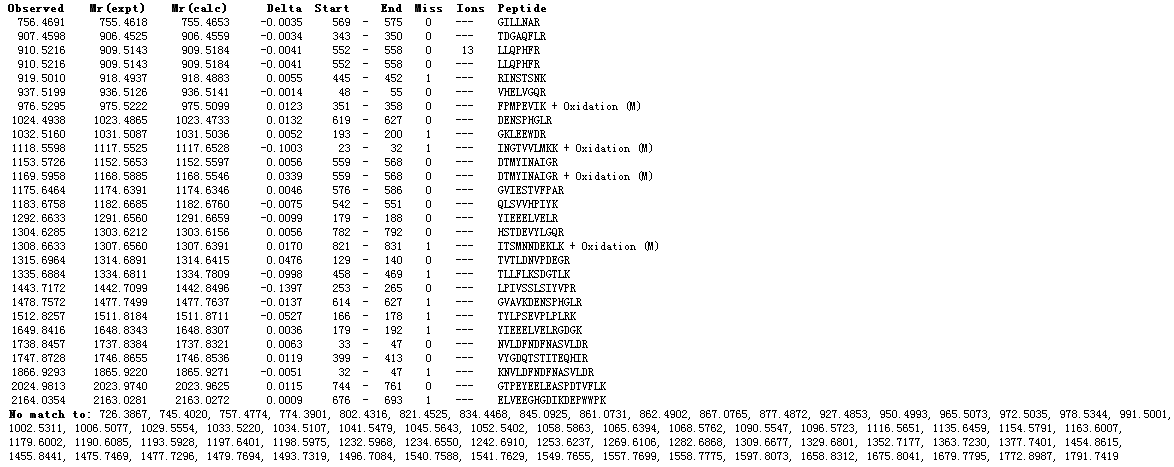
**

**β-galactosidase:**

**Mass-spectrum (MS/MS):**

**
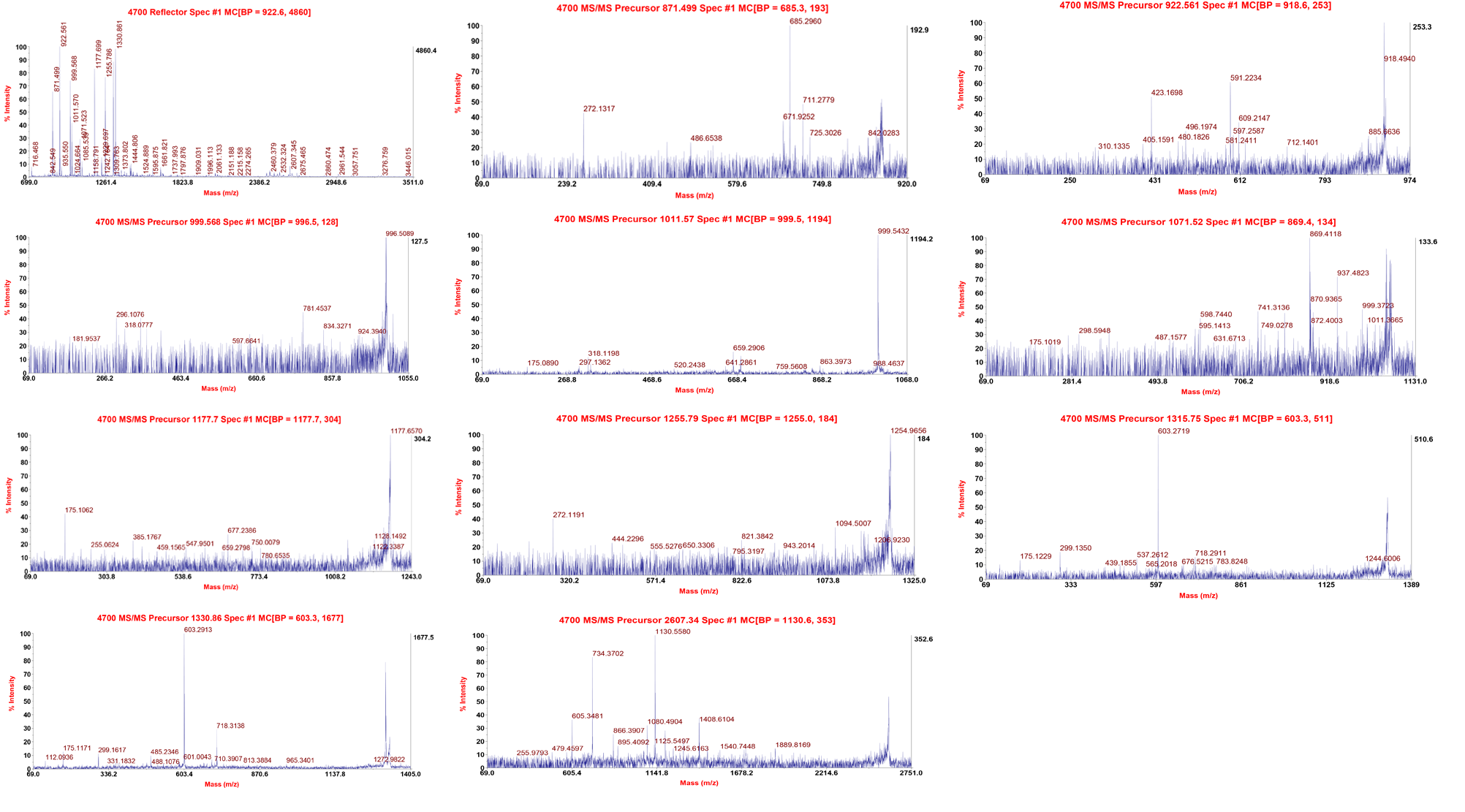
**

**Identification:**

**gi|507278 Mass:81628 Score:232 Expect: 2.3e-020 Matches: 43**

b-galactosidase-related protein; putative [Malus domestica]

**
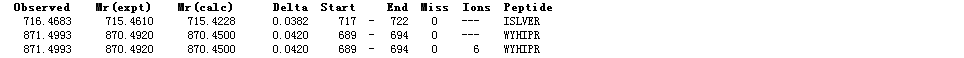
**

**
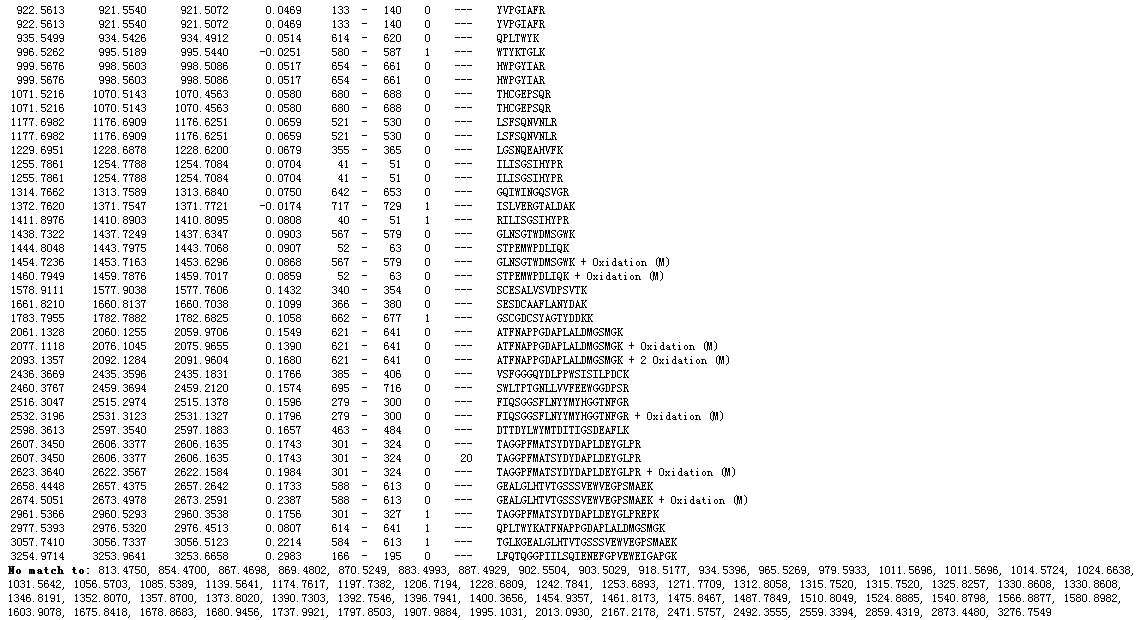
**

**NADP-ME:**

**Mass-spectrum (MS/MS):**

**
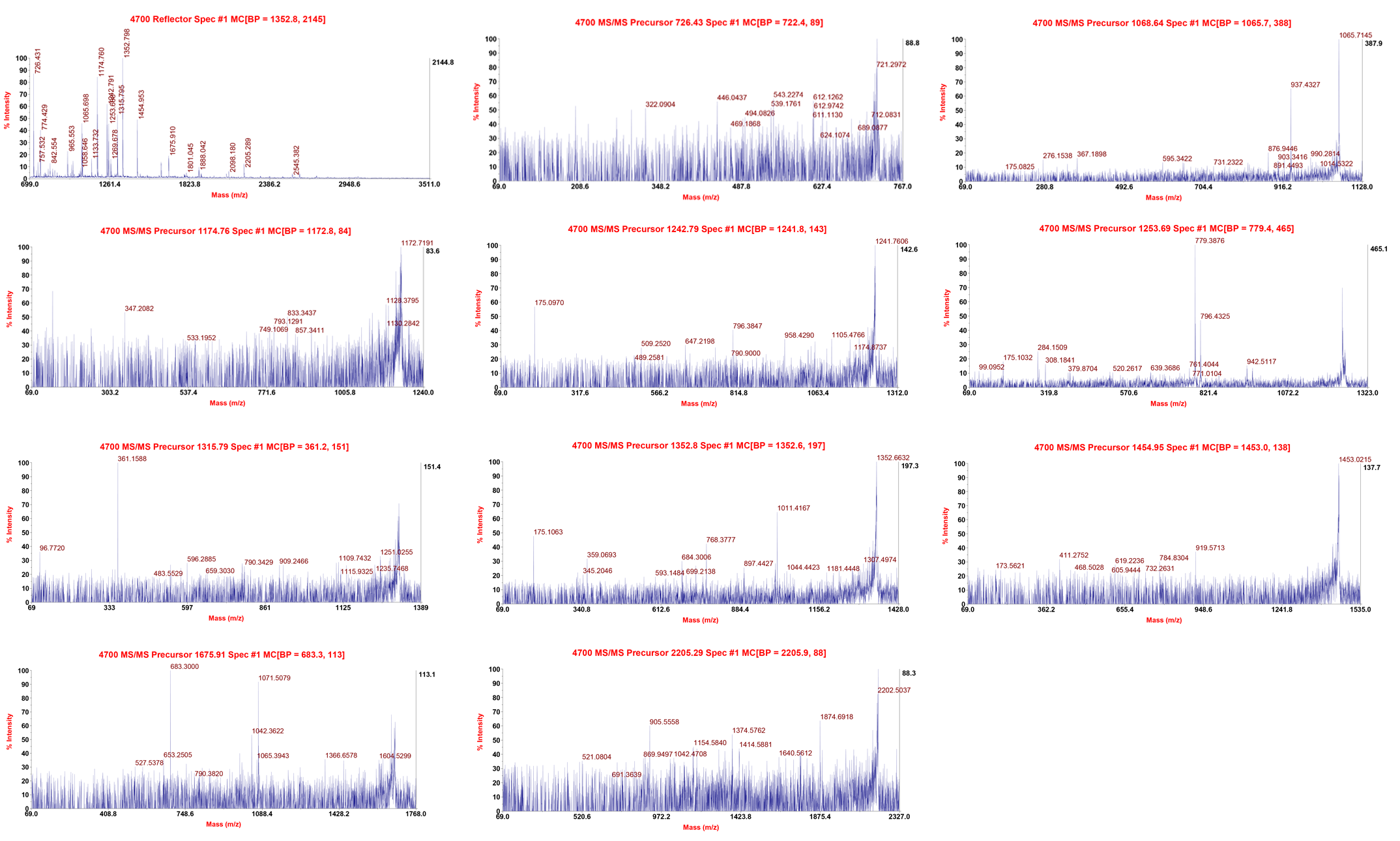
**

**Identification:**

**gi|470102042 Mass:65317 Score:96 Expect: 9.1e-005 Matches: 20**

PREDICTED: NADP-dependent malic enzyme-like [Fragaria vesca subsp. vesca]


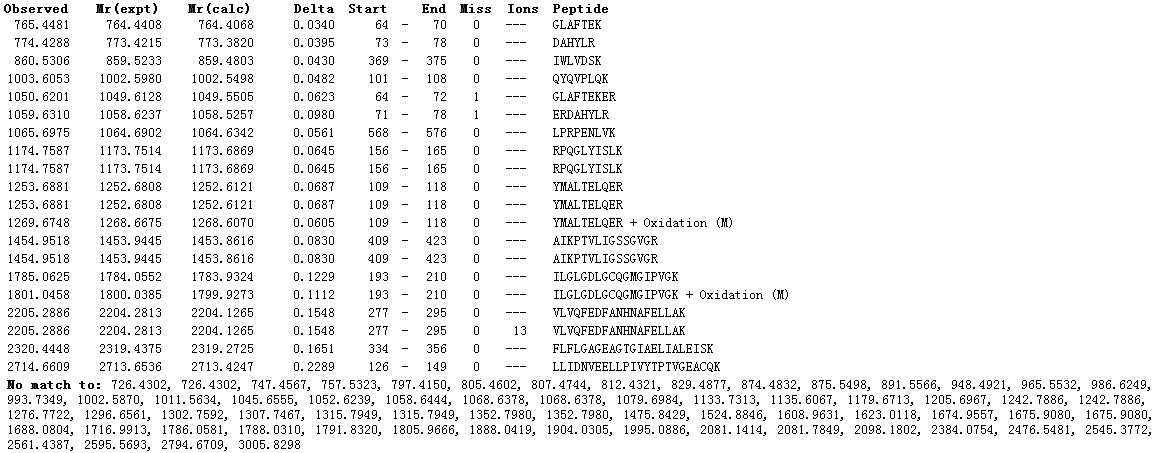


**dehydrin:**

**Mass-spectrum (MS/MS):**

**
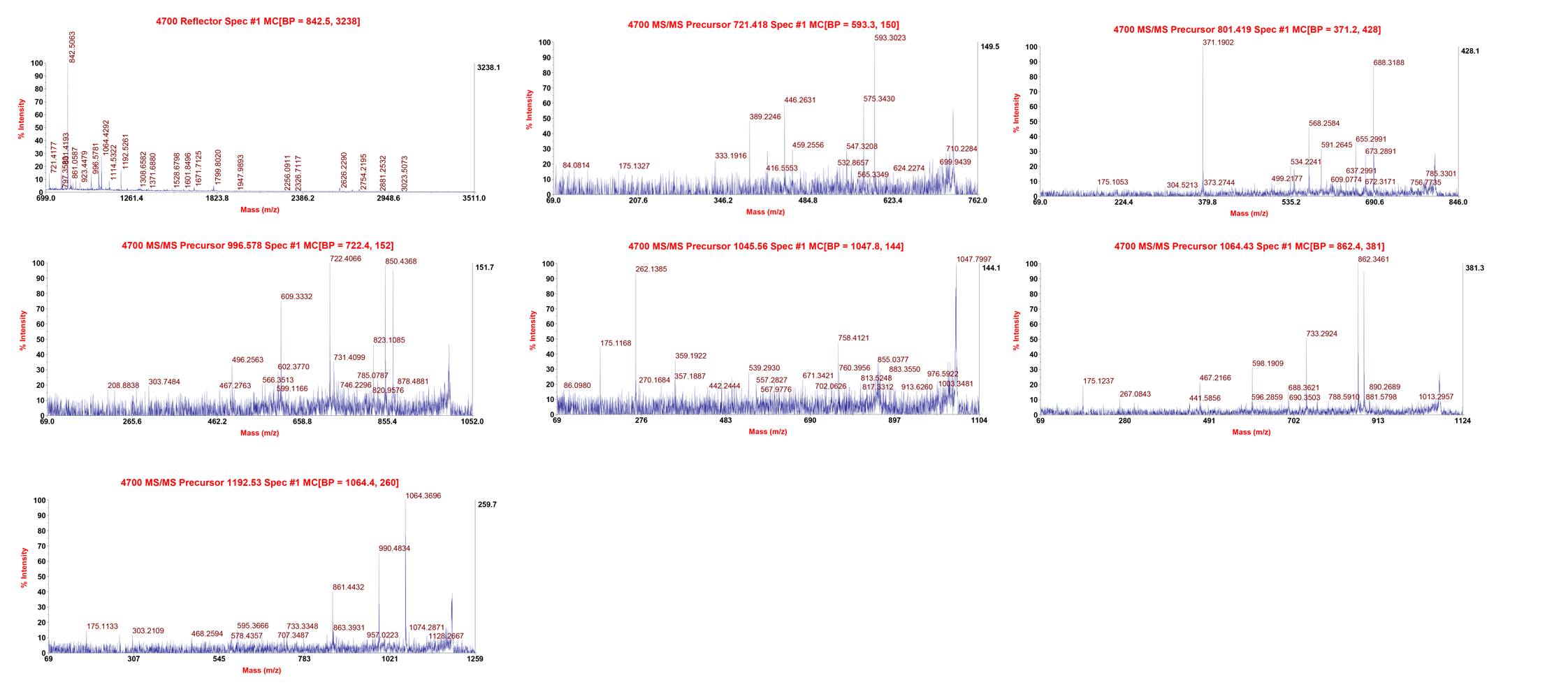
**

**Identification:**

**gi|658033116 Mass:31783 Score:297 Expect: 4.7e-025 Matches: 18**

PREDICTED: dehydrin COR47-like [Malus domestica]

**
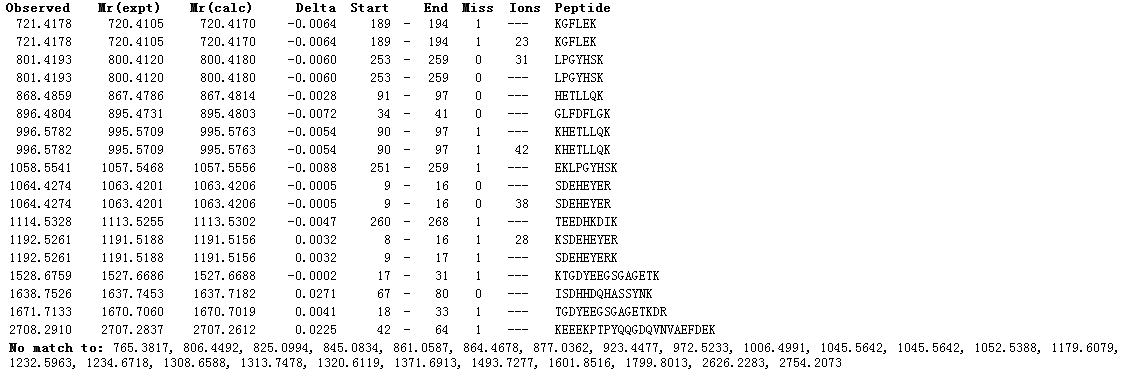
**

**UDP-glucose pyrophosphorylase:**

**Mass-spectrum (MS/MS):**

**
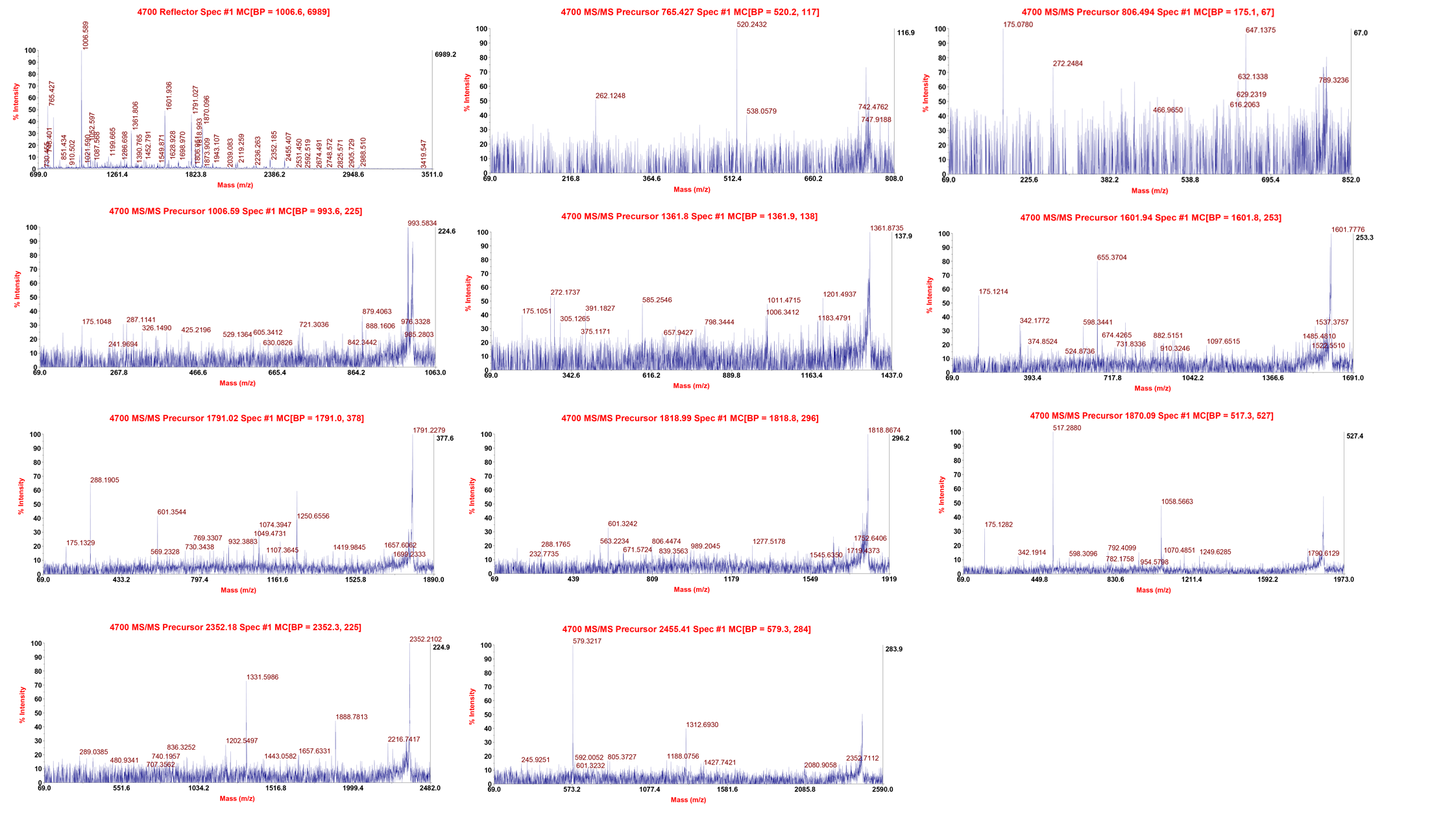
**

**Identification:**

**gi|3107937 Mass:51984 Score:83 Expect: 0.00036 Matches: 16**

UDP-glucose pyrophosphorylase [Pyrus pyrifolia]

**
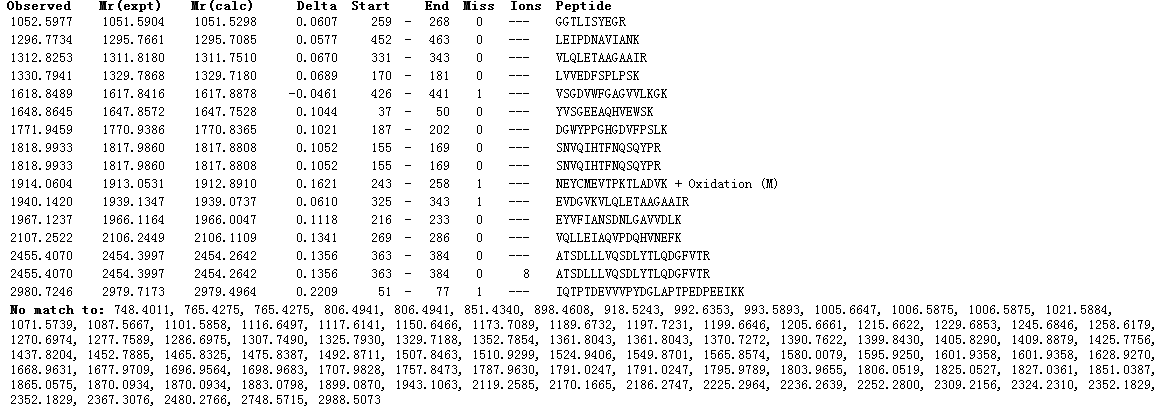
**

**phosphoglycerate kinase:**

**Mass-spectrum (MS/MS):**

**
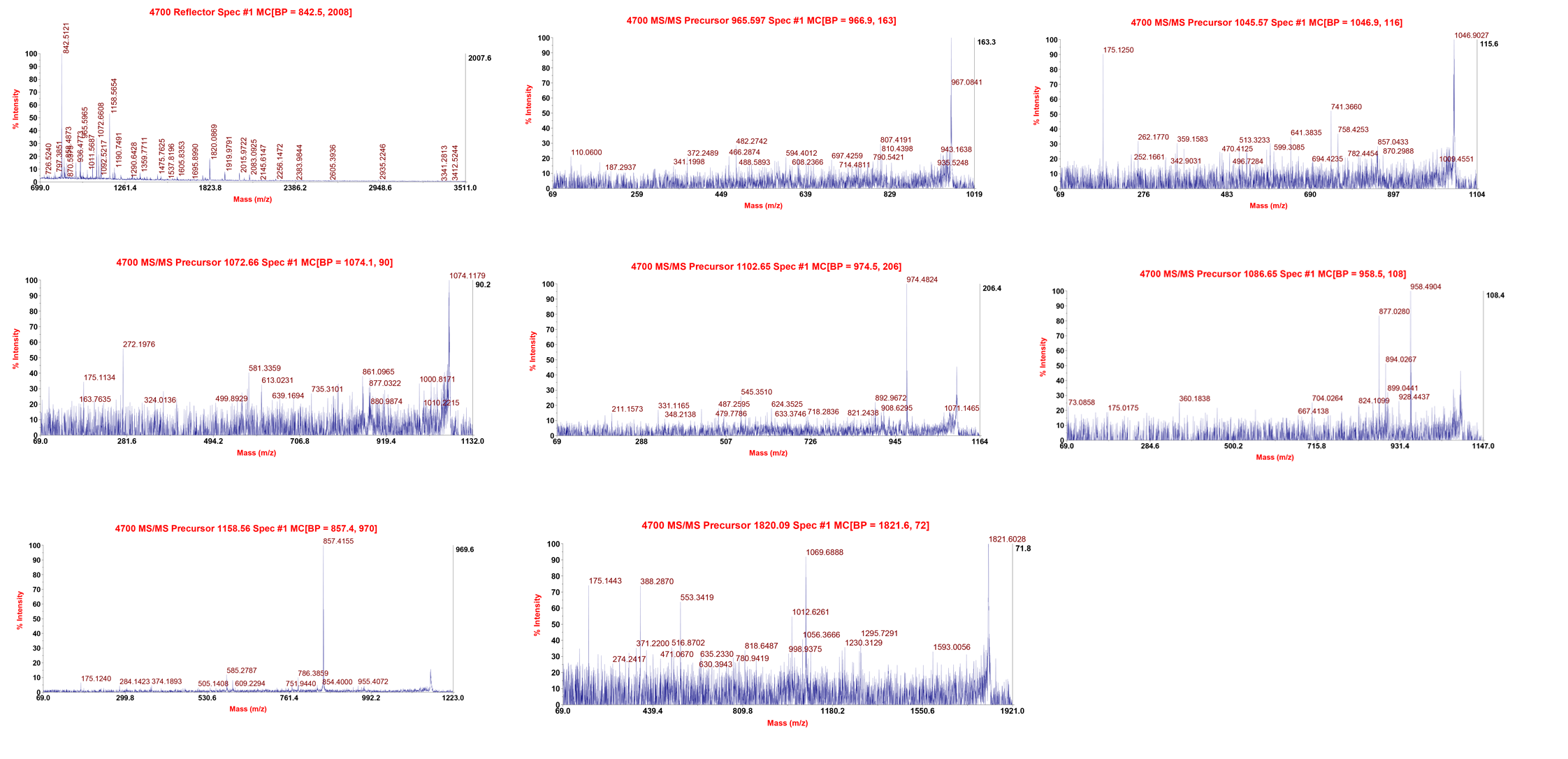
**

**Identification:**

**gi|657982239 Mass:42392 Score:170 Expect: 2.4e-012 Matches: 16**

PREDICTED: phosphoglycerate kinase, cytosolic [Malus domestica]**
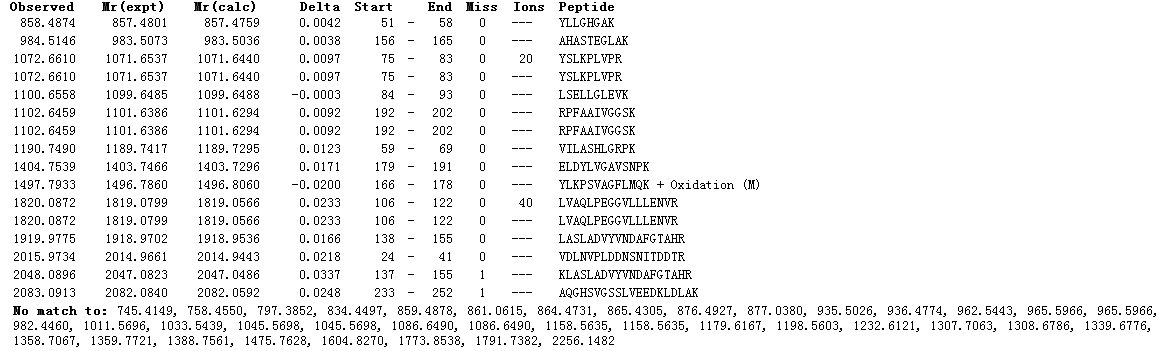
**

**glutamine synthetase:**

**Mass-spectrum (MS/MS):**

**
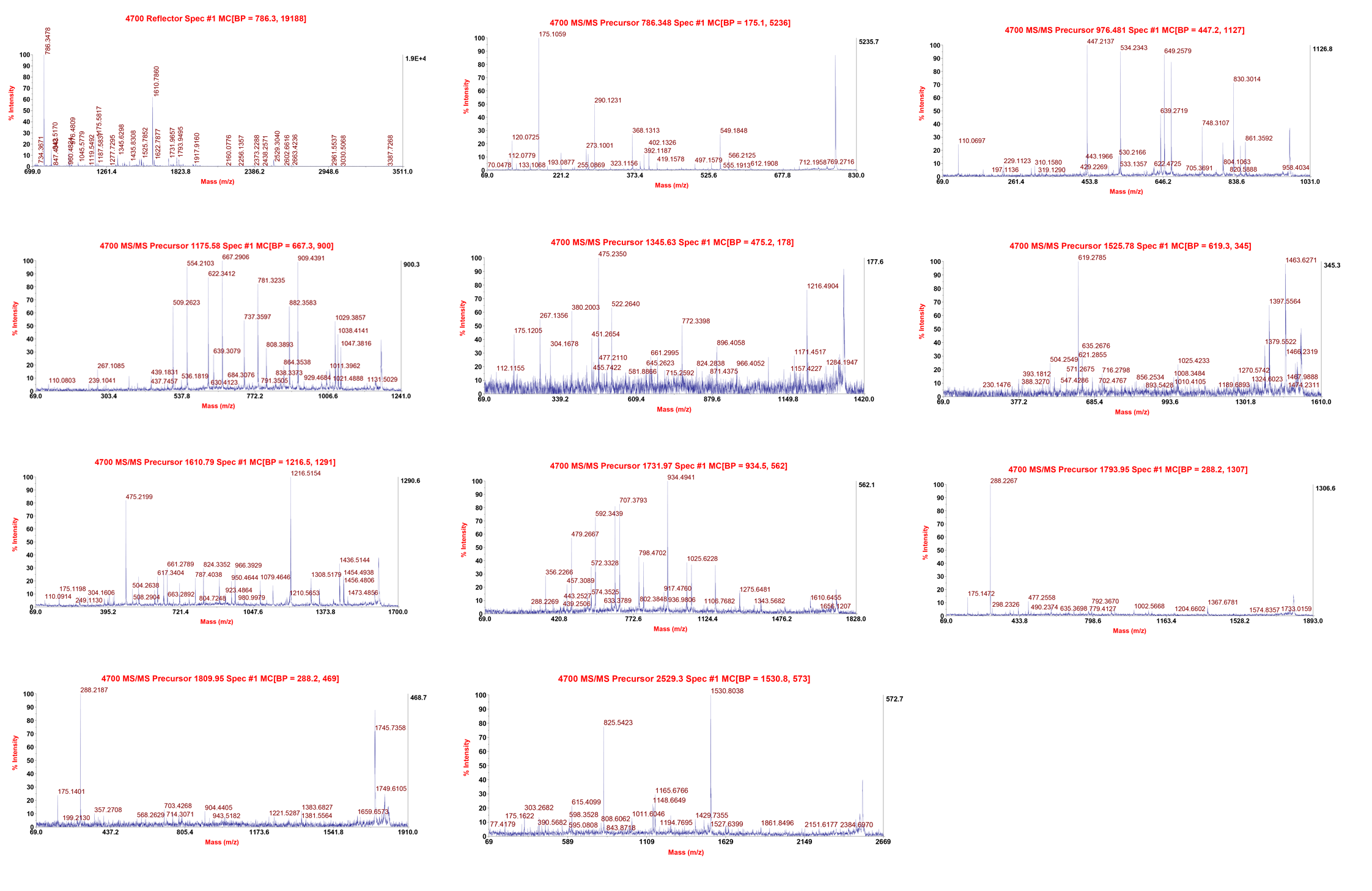
**

**Identification:**

**gi|658036616 Mass:39096 Score:650 Expect: 2.4e-060 Matches: 30**

PREDICTED: glutamine synthetase cytosolic isozyme [Malus domestica]

**
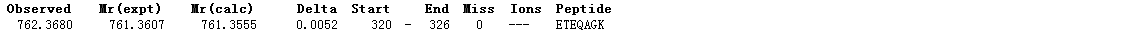
**

**
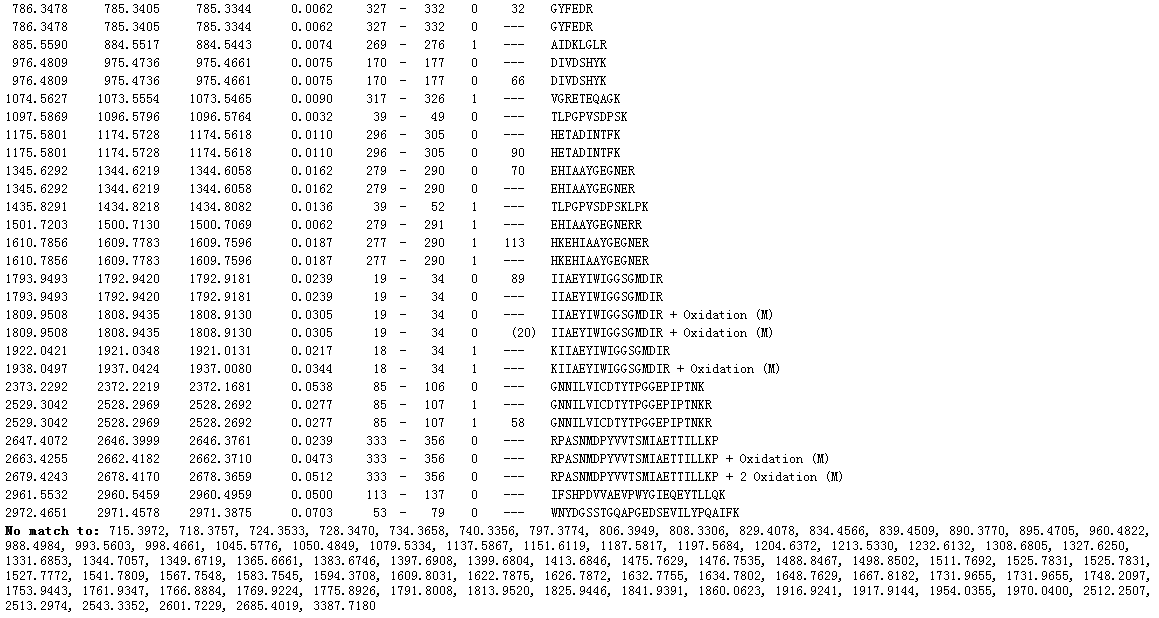
**

**thaumatin-like protein precursor:**

**Mass-spectrum (MS/MS):**

**
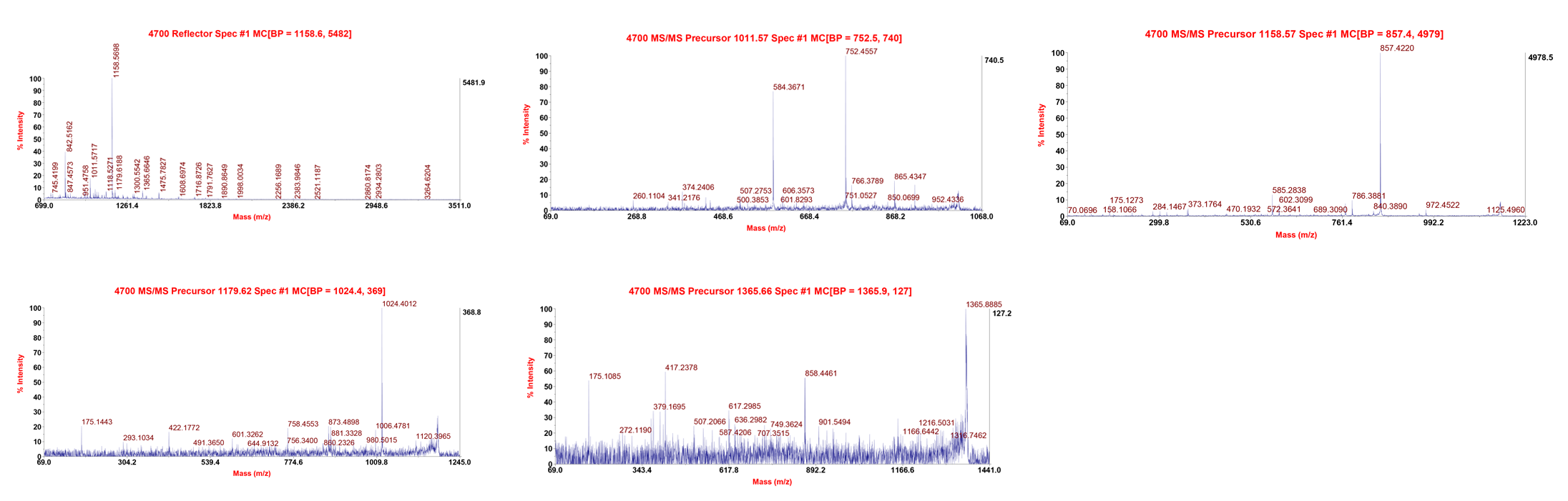
**

**Identification:**

**gi|3643249 Mass:26609 Score:160 Expect: 2.4e-011 Matches: 8**

thaumatin-like protein precursor Mdtl1 [Malus domestica]

**
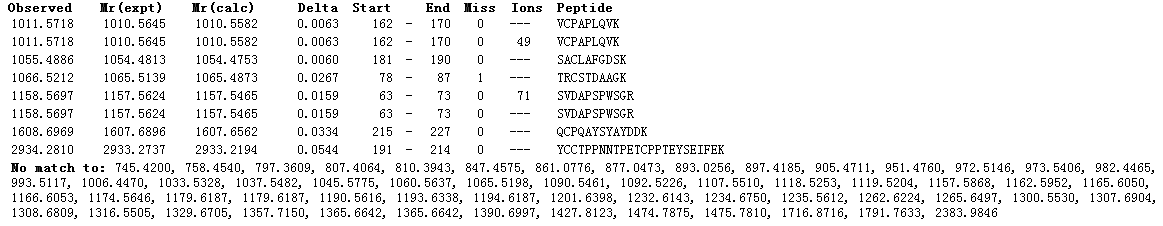
**

**thaumatin-like protein:**

**Mass-spectrum (MS/MS):**

**
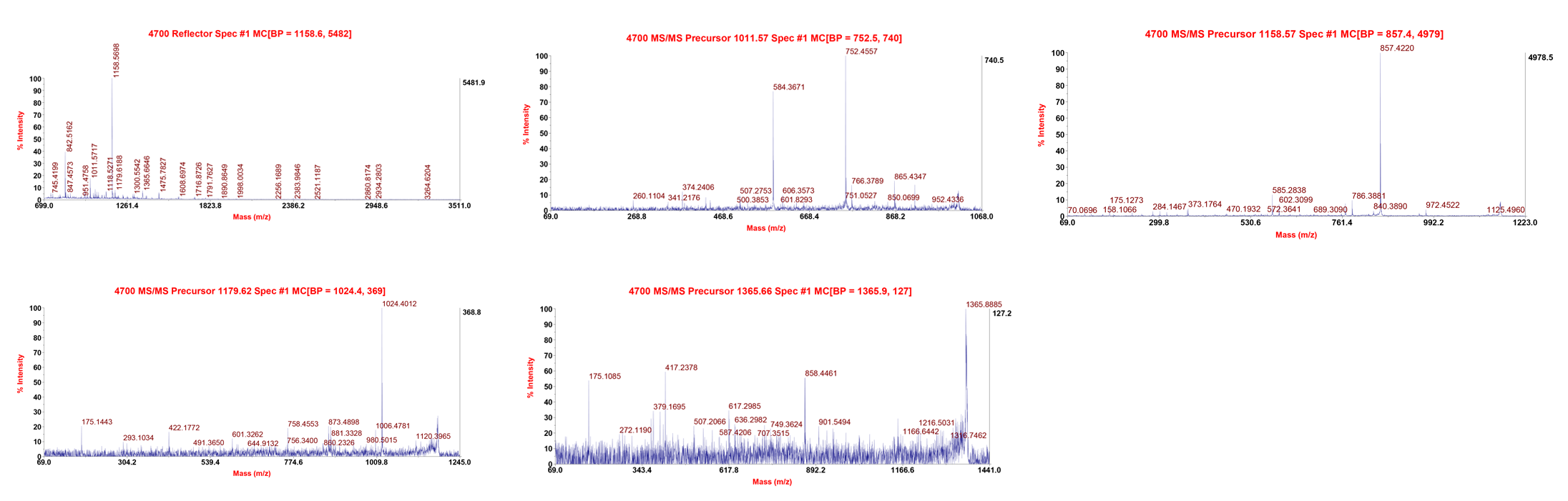
**

**Identification:**

**gi|394986175 Mass:24108 Score:113 Expect: 3.6e-007 Matches: 10**

Chain A, High Resolution Structure Of Mal D 2, The Thaumatin Like Food Allergen From Apple

**
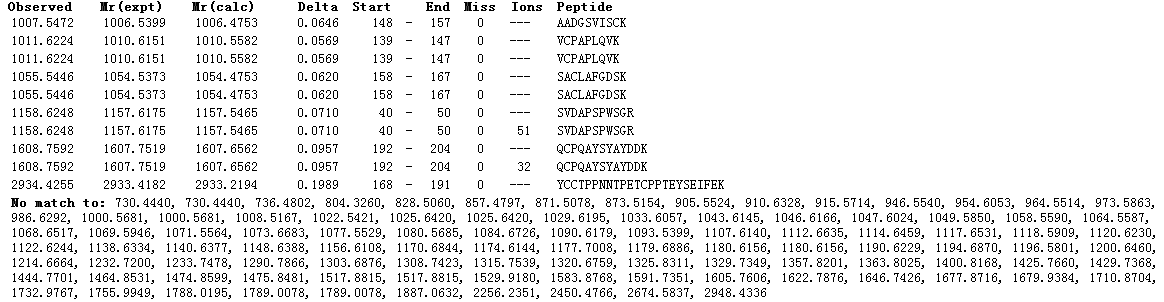
**

**exoribonuclease**

**Mass-spectrum (MS/MS):**

**
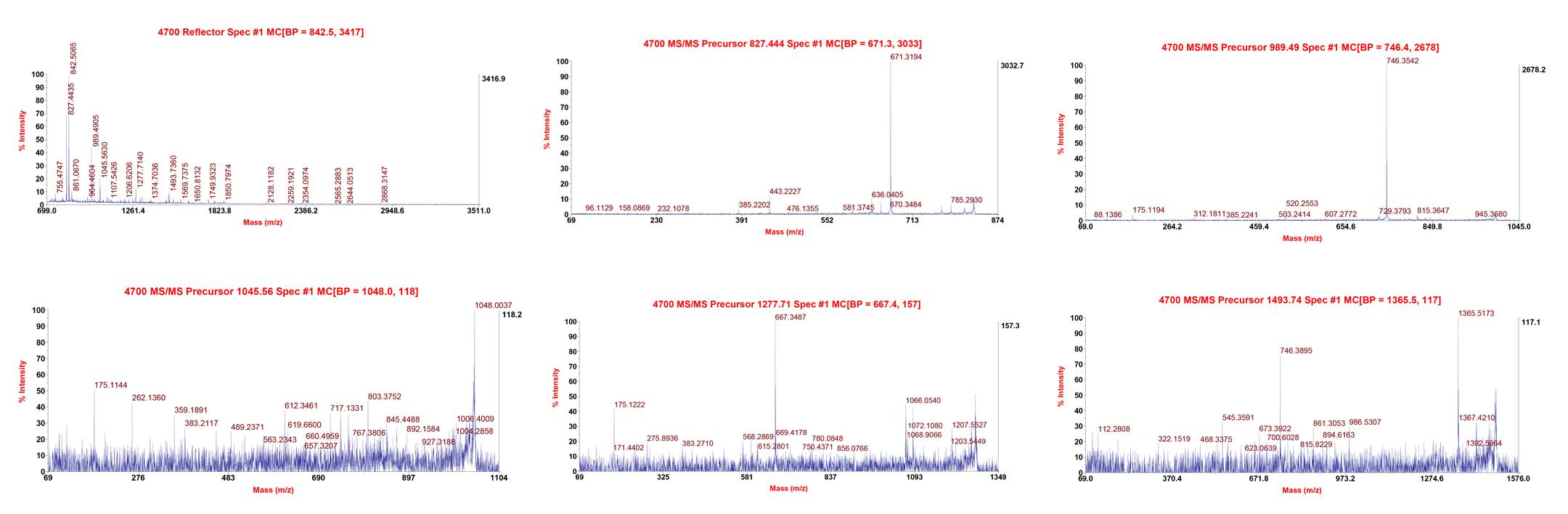
**

**Identification:**

**gi|657979046 Mass:22112 Score:86 Expect: 0.00067 Matches: 6**

PREDICTED: 5'-3' exoribonuclease 2-like [Malus domestica]

**
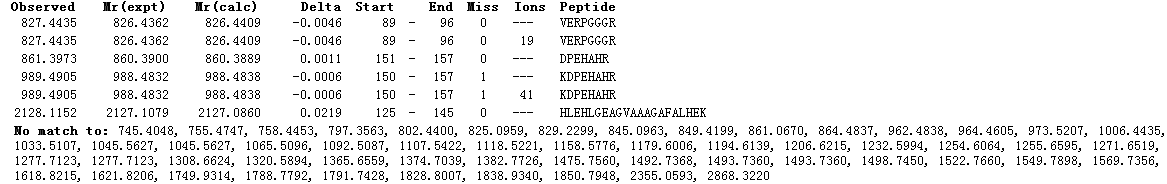
**

**ferritin:**

**Mass-spectrum (MS/MS):**

**
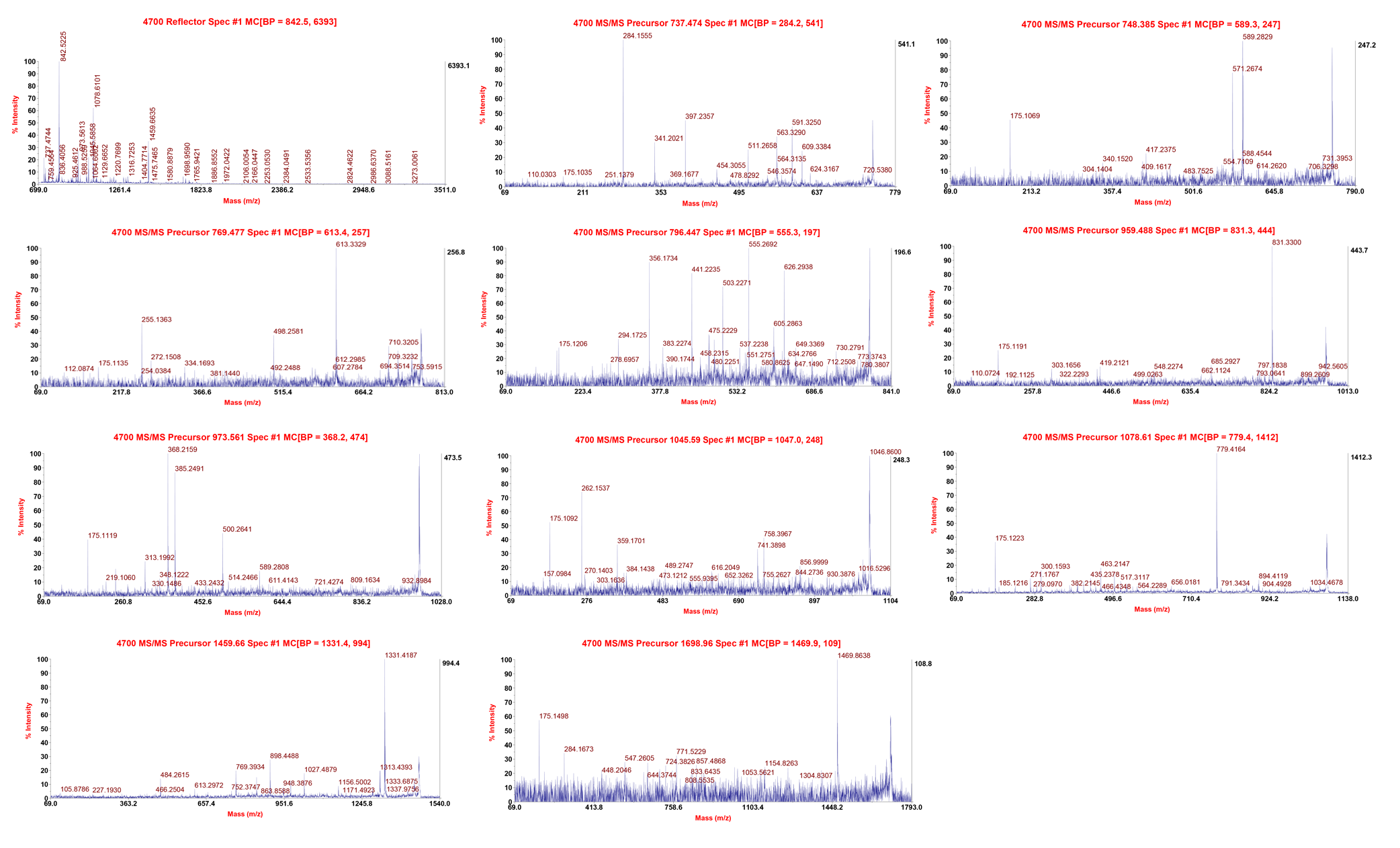
**

**Identification:**

**gi|657992077 Mass:29414 Score:252 Expect: 1.5e-020 Matches: 19**

PREDICTED: ferritin-4, chloroplastic-like [Malus domestica]

**
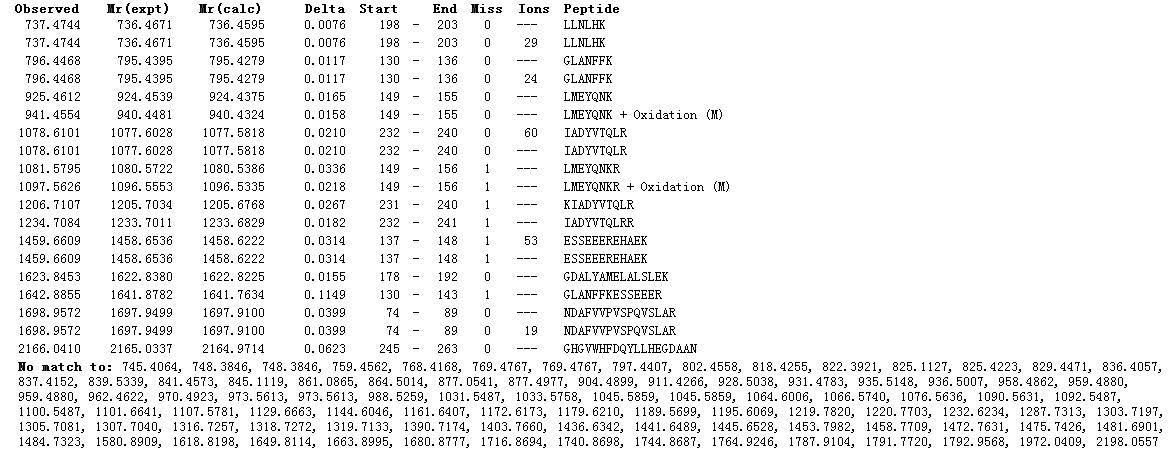
**
